# The Selection and Validation of Reference Genes for mRNA and microRNA Expression Studies in Human Liver Slices Using RT-qPCR

**DOI:** 10.3390/genes10100763

**Published:** 2019-09-28

**Authors:** Tomáš Zárybnický, Petra Matoušková, Martin Ambrož, Zdeněk Šubrt, Lenka Skálová, Iva Boušová

**Affiliations:** 1 Department of Biochemical Sciences, Charles University, Faculty of Pharmacy in Hradec Králové, 500 05 Hradec Králové, Czech Republic; zarybnto@faf.cuni.cz (T.Z.); matousp7@faf.cuni.cz (P.M.); ambrozm@faf.cuni.cz (M.A.); skaloval@faf.cuni.cz (L.S.); 2 Department of General Surgery, Third Faculty of Medicine and University Hospital Královské Vinohrady, Charles University, 100 34 Prague, Czech Republic; subrt@email.cz; 3 Department of Surgery, University Hospital Hradec Králové, 500 05 Hradec Králové, Czech Republic

**Keywords:** human liver, precision-cut liver slices, reference gene, RT-qPCR, miRNA, mRNA

## Abstract

The selection of a suitable combination of reference genes (RGs) for data normalization is a crucial step for obtaining reliable and reproducible results from transcriptional response analysis using a reverse transcription-quantitative polymerase chain reaction. This is especially so if a three-dimensional multicellular model prepared from liver tissues originating from biologically diverse human individuals is used. The mRNA and miRNA RGs stability were studied in thirty-five human liver tissue samples and twelve precision-cut human liver slices (PCLS) treated for 24 h with dimethyl sulfoxide (controls) and PCLS treated with β-naphthoflavone (10 µM) or rifampicin (10 µM) as cytochrome P450 (CYP) inducers. Validation of RGs was performed by an expression analysis of *CYP3A4* and *CYP1A2* on rifampicin and β-naphthoflavone induction, respectively. Regarding mRNA, the best combination of RGs for the controls was *YWHAZ* and *B2M*, while *YWHAZ* and *ACTB* were selected for the liver samples and treated PCLS. Stability of all candidate miRNA RGs was comparable or better than that of generally used short non-coding RNA U6. The best combination for the control PCLS was miR-16-5p and miR-152-3p, in contrast to the miR-16-5b and miR-23b-3p selected for the treated PCLS. Our results showed that the candidate RGs were rather stable, especially for miRNA in human PCLS.

## 1. Introduction

Precision-cut liver slices (PCLS) are a three-dimensional multicellular model with preserved tissue architecture as well as cell–cell and cell–matrix interactions that maintain and mimic liver in vivo functions. [1,2] The applicability of PCLS includes toxicity, inflammation and oxidative stress [3,4,5,6], and fibrosis as well as expression changes upon short-term or prolonged incubation of PCLS [7,8,9,10,11,12]. 

Choosing suitable reference genes (RGs) for the normalization of gene expression is always a dilemma. Reverse transcription-quantitative polymerase chain reaction (RT-qPCR) is a very potent tool with high sensitivity, accuracy, specificity, reproducibility, and relatively low cost in comparison to microarrays or high-throughput sequencing [13]. Nevertheless, RT-qPCR can be influenced by factors such as RNA integrity, genomic DNA contamination, reverse transcription reaction efficiency, and complementary DNA (cDNA) quality [14]. There have been no studies to properly validate the stability of RGs despite the spreading usage of PCLS, although PCLS are a useful model to study liver injury as well as repair processes, which are relevant for defining the dose-response profile of drug-induced injury, along with the identification of novel therapeutic targets [15]. 

A number of prerequisites are involved in searching for suitable and effective RGs to elicit reproducible results from any RT-qPCR analysis including a stable expression among the analyzed samples as well as preventing the samples from being affected by the experimental conditions [13,16,17]. The perfect reference gene does not exist, therefore using multiple validated genes is recommended to avoid variations due to technical or experimentally-induced issues [13,18]. The purpose of the present study was to render an independent recommendation of suitable RGs for the normalization of mRNA and microRNA (miRNA) in the RT-qPCR analysis of human PCLS from clinically heterogeneous samples (e.g., age, gender, malignancy, treatment regimen) [19].

## 2. Materials and Methods

### 2.1. Chemicals and Reagents

All chemicals were purchased from Sigma Aldrich (Prague, Czech Republic) unless otherwise stated. 

### 2.2. Ethics Statement

The Ethics Committee of University Hospital in Hradec Králové, Czech Republic (Permission No. 201703 S14P, 2 March 2017) approved all of the procedures, and all methods were performed in accordance with the relevant guidelines and regulations. All subjects included in this study signed an informed consent for tissue obtaining for research purposes.

### 2.3. Human Liver

Liver tissue was obtained from thirty-five patients (19 males and 16 females, 26–84 years old, Caucasian ethnicity) undergoing a partial hepatectomy (Table 1). Small pieces of the liver tissue were immediately put into an ice-cold vessel with Euro-Collins solution and immediately processed. These liver samples were regarded as healthy, based on the results of routine biochemical tests carried out prior to surgery (plasma levels of alanine aminotransferase (ALT), alkaline phosphatase (ALP), aspartate aminotransferase (AST), bilirubin (BIL), and γ-glutamyltransferase (GMT)) as well as the results of histological scoring of liver steatosis and fibrosis performed by a pathologist.

As not all liver samples were suitable for PCLS preparation, small pieces of twenty-three liver samples were only placed into TRI Reagent solution (Biotech, Prague, Czech Republic) and stored in a freezer at −80 °C until RNA isolation. Twelve liver samples were used for PCLS preparation according to the below mentioned protocol.

### 2.4. Preparation of Precision-Cut Liver Slice and Experimental Treatment

The PCLS were prepared according to Zárybnický et al. [4]. Briefly, cylindric cores were cut out of the tissue. A Krumdieck tissue slicer MD4000 (Alabama Research and Development, Munford, AL, USA) filled with a supplemented ice-cold Krebs–Henseleit buffer saturated with carbogen (95% O_2_, 5% CO_2_) was used to prepare the PCLS (thickness: 150 µm, diameter: 8 mm). The incubation of PCLS proceeded individually in 12-well plates filled with supplemented Williams’ Medium E with GlutaMAX (25 mM glucose and 50 µg/mL gentamycin; ThermoFisher, Waltham, MA, USA) at 37 °C under continuous supply of 85% O_2_/5% CO_2_ (Binder CB 60 incubator, Tuttlingen, Germany) with continuous shaking (90 times/min). All experiments were performed in triplicate. The slices were treated with 0.1% DMSO (controls), 10 µM rifampicin (RIF, a strong CYP3A4 inducer), or 10 µM β-naphthoflavone (BNF, a CYP1A1/2 inducer) [19].

### 2.5. Viability

Viability of the PCLS was measured in triplicate at multiple time points: −1 (before pre-incubation), 0 (after pre-incubation), 4, 8, 12, 18, and 24 h. The levels of adenosine triphosphate (ATP) and leakage of lactate dehydrogenase (LDH) were used as viability markers. The ATP levels were determined by the method by Hadi et al. [20], according to the protocol described in our previous report [4]. 

The LDH leakage into the culture medium, expressed as % of total LDH activity, was determined as a marker of plasma membrane damage. For the analysis, all of the culture medium was taken up and kept in −80 °C until use. After thawing, the medium was centrifuged at 2000 rpm for 5 min at 4 °C to remove cellular debris. To determine the total LDH activity, three slices were incubated for 24 h, and afterward, each slice was homogenized in its culture medium by ribbed shaft pestles, with these homogenates processed in the same way as the tested culture mediums. LDH activity was determined using the LDH cytotoxicity assay (Roche Diagnostics, Mannheim, Germany). The absorbance of technical duplicates was measured using the plate reader Infinite M200 (Tecan Group, Mannedorf, Switzerland). 

### 2.6. Tissue RNA Extraction

The PCLS were collected after 4, 8, 12, 18, and 24 h. All treatments were performed in triplicate and every slice was put separately into 500 μL of TRI Reagent and stored at −80 °C until use for at most up to two months. Total RNA isolation from each slice was performed according to the manufacturer’s instructions (TRI Reagent®, Biotech, Prague, Czech Republic). The sample homogenization was carried out using a single metal bead in a 2 mL Eppendorf tube using a microhomogenizer. Forty µL of diethyl pyrocarbonate (DEPC)-treated water (0.01% DEPC in HPLC water, autoclaved) was used to dissolve the purified RNA, which was then stored at −80 °C. RNA yields and purity were evaluated by measuring the absorbance at 260 and 280 nm using a NanoDrop ND-1000 UV–Vis Spectrophotometer (Thermo Fisher Scientific, Prague, Czech Republic), where all samples had a ratio of absorbance at 260 nm and 280 nm higher than 1.8. RNA integrity numbers (RIN) were detected by an Agilent 2000 analyzer (Agilent, Santa Clara, CA, USA), with the RIN of all samples greater than 6.0 (Table S1). Furthermore, DNAse treatment was performed to avoid genomic DNA contamination. Details of this procedure are described in Table S2. The DNAse I treated RNA was stored at −80 °C until further analyses. 

### 2.7. cDNA Synthesis

Details of cDNA synthesis for mRNA, miRNA, and U6 are summarized in Table S2. For mRNA and miRNA, the obtained cDNAs were diluted 1:6 and 1:9 with DEPC water, respectively. All cDNAs were stored at −20 °C until the qPCR assay.

### 2.8. Primer Design, Quantitative Real-Time PCR

Descriptions of selected candidate RGs for normalization of mRNA and miRNA as well as genes of interest are mentioned in Table 2. For mRNA normalization, the primers were either described in previous reports or designed manually using a Primer3 software [21] targeting sequence outside the hairpin structures calculated for 60 °C using mFold [22], with the specificity checked using NCBI primer blast. The reverse primer used for miRNA normalization was universal (the sequence inserted by the Stem-Loop primer) [23,24] and the forward primers were designed manually and controlled using OligoCalc [25]. Synthesis of all primers was carried out by Generi Biotech (Hradec Králové, Czech Republic). The primer sequences and other information are also listed in Table 2. Melting curve plots are presented in Supplementary Materials (Figure S1). 

RT-qPCR was performed following the minimum information for publication of quantitative real-time PCR experiments (MIQE) guidelines [17] and all experimental details are stated in Table S2.

### 2.9. Data Analysis

Since the PCLSs from each human were processed separately, each analysis was performed individually. Two types of software were used to assess the stability of the candidate RGs, and the analyses were based on untransformed Ct values. RefFinder is a freely available webtool, the main advantage of which is its ability to integrate four computational algorithms (geNorm [13], BestKeeper [26], NormFinder [27], and Comparative ΔCt method [28]) [29]. RefFinder ranking of the genes is based on the geometric mean of placings from each of the four above-mentioned programs [29]. Although the stability ranking of the candidate genes is important, it does not provide information regarding how many reference genes are necessary for normalization. Therefore, we additionally used the freely available Microsoft Excel geNorm version (inputting the transformed Ct values into relative quantities) to estimate the optimal number of RGs that should be used for the intended experiment via the calculation of pairwise variation values [13] (the geNorm output is not included in RefFinder). 

Relative expression levels of the target genes were calculated as the fold change from the triplicates in each group using the 2^-ΔΔCt^ method [30]. Results were expressed as the mean ± SD. Comparisons among the control and treated groups were performed using a two-way analysis of variance (ANOVA), using GraphPad Prism 7 (GraphPad Software, La Jolla, CA, USA). Differences were considered significant at *p* < 0.05.

## 3. Results

All liver samples included in this study were selected to be as healthy as possible even if patients were being treated under different clinical conditions. Based on the steatosis (score of 0–1) and fibrosis (score of zero) scoring performed by a pathologist, all the liver biopsies showed no or mild signs of liver disease. As biochemical markers of liver functions, plasma levels of BIL, ALT, AST, GMT, and ALP were assessed (Table S3). All of the assessed biochemical parameters were within physiological ranges in thirteen samples, while two samples (L6 and L23) exerted an elevated BIL level, probably as a result of intrahepatic obstruction due to a malignancy. ALP only showed an increase in four samples, with GMT only elevated in twelve samples; a combination of ALP-GMT and AST-GMT was elevated in two samples and one sample, respectively. Moreover, plasma levels of ALT, AST, GMT, and ALP were increased in L21 and L37 with an AST/ALT ratio of 0.62 and 0.59, respectively. Routinely tested liver biomarkers (e.g., ALT, AST, GMT, BIL) are altered in different malignities and hepatic metastases as well as in patients with primary or secondary malignant involvement of the hepatic system, all of which represent significant risk predictors for all-cause mortality [31]. 

### 3.1. Analysis of Candidate RGs Expression Stability for mRNA Normalization and Their Validation in Human Liver Tissue

**Figure 1:**
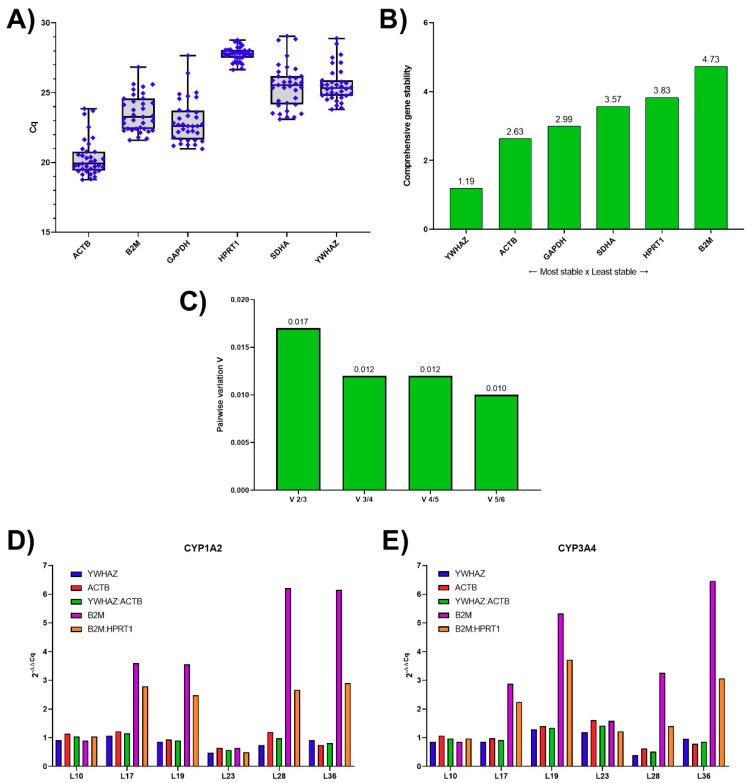
Analysis of candidate reference genes for mRNA normalization in human liver. (**A**) Boxplot of quantification cycle (Cq) values for each RG in all liver samples (*n* = 35). The box indicates the 25% and 75% percentiles, whiskers represent the maximum and minimum values and the median is depicted by the line across the box; (**B**) Comprehensive stability values of RGs according to RefFinder; (**C**) Determination of the optimal number of reference genes (RGs) by geNorm analysis; (**D**, **E**) Effects of different normalization approaches on the expression of CYP1A2 (**D**) and CYP3A4 (**E**).

With regard to the information obtained from literature concerning RGs in liver/PCLS, a set of six candidate RGs, namely beta-actin (*ACTB*), beta-2-microglobulin (*B2M*), glyceraldehyde 3-phosphate dehydrogenase (*GAPDH*), hypoxanthine phosphoribosyltransferase 1 (*HPRT1*), subunit A of succinate dehydrogenase complex (*SDHA*), and tyrosine 3-monooxygenase/tryptophan 5-monooxygenase activation protein zeta (*YWHAZ*) was tested in human liver samples collected from patients undergoing partial hepatectomy [3,7,9,10,32,33]. The raw quantification cycle (Cq) values were obtained for all thirty-five human liver tissue samples (Figure 1A). In this study, *HPRT1* was the candidate RG with the lowest expression (mean Cq = 27.76, SD = 0.50) as well as the lowest variability (coefficient of variation, CV = 1.80%), while *ACTB* was the most abundantly expressed gene (mean Cq = 20.36, SD = 1.34). The highest variability was found for *GAPDH* with CV = 6.69%.

The ranking of the expression stability of RGs was performed using RefFinder [29], which integrates four computational algorithms to compare and rank the candidate RGs. Based on the rankings from each program, RefFinder assigns a corresponding weight to an individual gene and calculates the geometric mean of their weights for the overall final ranking. Moreover, the ranking of RGs based on their expression stability measure (M), which represents the average pairwise variation of a certain gene with all other tested reference genes [13], was performed using geNorm. The most stably expressed RGs in the human liver samples were *YWHAZ* and *ACTB*, with an M value of 0.742 (Figure 1B). The raw data from RefFinder are presented in Table S4.

In addition, geNorm enables the calculation of the pairwise variation (V_n_/V_n+1_) between two sequential candidate RGs to define the optimal number of RGs that should be used. Vandesompele et al. (2002) defined a pairwise variation of 0.15 as the cut-off value, which indicates that the inclusion of an additional RG is needless [13]. In our analysis, the V2/3 value was 0.017, suggesting two RGs as optimal (Figure 1C).

The use of different RGs to calculate the relative expression data of target genes could significantly affect the final normalized results. To show the impact of different RGs on the result of the practical experiment, the relative expression patterns of *CYP1A2* and *CYP3A4* were analyzed, with differences shown for six selected liver samples (Figure 1D,E, rest in Table S5). Different normalizations were applied: (1) the most stable gene (*YWHAZ*); (2) the second most stable gene (*ACTB*); (3) the geometric mean of *YWHAZ* and *ACTB*; (4) the least stable gene (*B2M*); and (5) the geometric mean of two of the least stable genes (*B2M*/*HPRT1*). In all liver samples, the expression levels of both CYPs were comparable when the three most stable normalizations (1–3) were applied. Moreover, the way of normalization did not influence the expression level of the target genes in some of the liver samples (e.g., L10 and L23). However, when *CYP1A2* and *CYP3A4* were normalized using normalizations 4–5, the expression levels of both CYPs increased several times when compared to normalizations 1–3 in many of the liver samples (e.g., L17, L19, L28, and L36). 

### 3.2. Analysis of Candidate RGs Expression Stability for mRNA Normalization in Human PCLS

Subsequently, results obtained with human liver tissue were compared to the human PCLS. Analysis of candidate RGs stability in human PCLS was performed in two ways. In the first part, PCLS obtained from the liver of twelve patients were incubated in the medium containing DMSO (controls) or the CYP inducers BNF and RIF (treated groups) for 24 h, following which the expression of RGs was assessed. In the second part, the mRNA expression of the RGs was monitored at five time points within a 24 h incubation of PCLS with DMSO or CYP inducers. Such an experiment is demanding with regard to the amount of the already rare tissue, therefore, only liver samples obtained from three individuals were used for this experiment.

**Figure 2:**
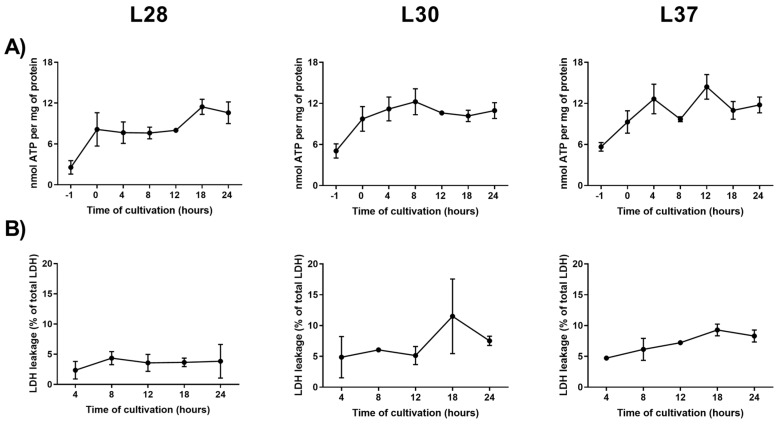
Viability results of human precision-cut liver slices (PCLS) during 24-h incubation. (**A**) adenosine triphosphate content; (**B**) lactate dehydrogenase leakage. Results are presented as mean ± SD (*n* = 3).

The first and crucial step in this experiment was to ensure optimal culture conditions for the PCLS, otherwise the expression rate of the tested genes could be unpredictably influenced. Levels of ATP and leakage of LDH were used as viability markers. ATP levels increased after pre-incubation, a finding which could be explained by the restoration of mitochondrial functions after the ischemic period during surgery. ATP levels remained rather stable for the rest of the experiment. At each time-point, LDH leakage usually did not exceed 10% of total LDH activity, which denotes that the plasma membranes of the liver cells were not significantly damaged (Figure 2).

### 3.3. Reference Genes Ranking According to Their Expression Stability

After performing RT-qPCR, the Cq values from the controls and treated groups were analyzed separately. Slices from each human sample were analyzed individually, since they were also processed and measured separately; in addition, potential biological variation can be shown this way. RefFinder was used to create an overall comprehensive ranking of the most stable RGs for our defined groups, with raw data shown in Table S6 and Table S7. Moreover, the V2/3 value was in all cases lower than 0.01, suggesting two RGs as optimal for normalization (Figures S2 and S3).

**Figure 3:**
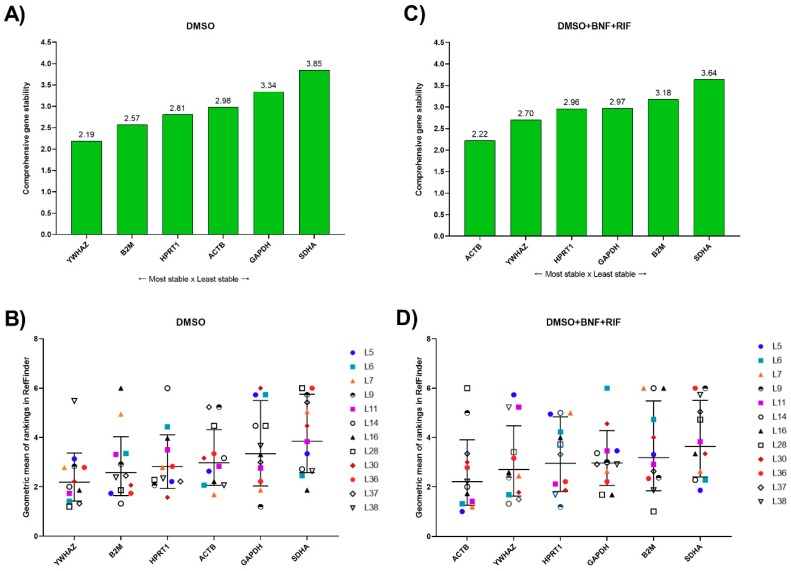
Analysis of candidate reference genes for mRNA normalization in human PCLS. (**A**) Comprehensive stability values of RGs in PCLS incubated for 24 h with dimethyl sulfoxide (DMSO) according to RefFinder. Genes are presented on the X-axis in the order of decreasing stability; (**B**) RefFinder ranking of RGs with individual values in PCLS incubated for 24 h with DMSO (controls); and (**C**) Comprehensive stability values of RGs in PCLS incubated for 24 h with cytochrome P450 (CYP) inducers (treated groups) according to RefFinder. Genes are presented on the X-axis in the order of decreasing stability; (**D**) RefFinder ranking of RGs with individual values in PCLS incubated for 24 h with CYP inducers (treated groups).

The best combination of RGs was selected based on the overall rankings of RGs in RefFinder for individual human samples. Optimally, the gene stability should be equal among the samples, however, biological variation plays an unpredictable role, and therefore we recommend a combination of genes for which the overall rankings were lowest among human samples. According to RefFinder, the most stable genes for 24 h incubated PCLS in the controls were *YWHAZ* and *B2M*, while *ACTB* and *YWHAZ* were the most stable genes in the treated groups (Figure 3A,C). It is important to emphasize that differences in stability of our tested RGs were small (rankings in Figure 3B,D).

**Figure 4:**
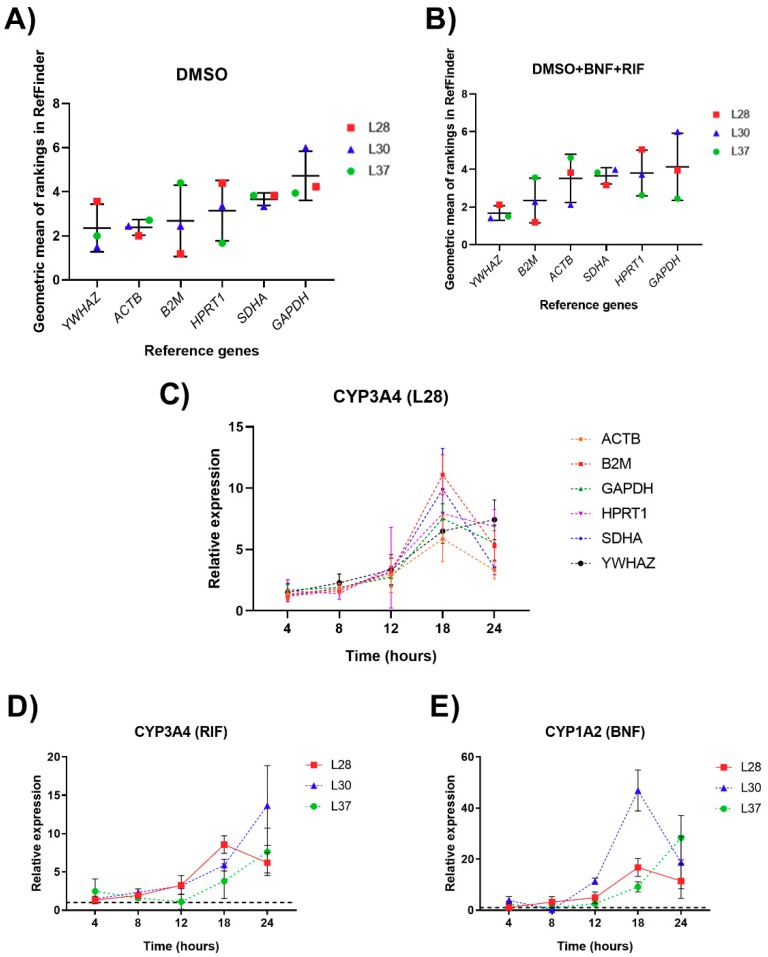
RefFinder ranking of RGs with individual values in PCLS within 24-h incubation in the control (**A**) and treated group (**B**); (**C**) Effects of different reference genes on the normalization of CYP3A4 expression in sample L28. Results are presented as mean ± SD (*n* = 3); (**D**,**E**) Relative mRNA expression of CYP3A4 (**D**) and CYP1A2 (**E**) under the effect of rifampicin (RIF) 10 µM and β-naphthoflavone (BNF) 10 µM, normalized to geometric mean of YWHAZ and B2M. Results are presented as mean ± SD (*n* = 3).

For the RGs’ expression stability within 24 h incubation, all the tested RGs showed a stable expression during incubation. In the controls, the stability order (shown in descending stability manner) was as follows: *YWHAZ* > *ACTB* > *B2M* > *HPRT1* = *SDHA* > *GAPDH*, while the stability order in the treated groups was *YWHAZ* > *B2M* > *ACTB* > *HPRT1* = *SDHA* = *GAPDH* (Figure 4A,B). GeNorm analysis showed that a combination of two RGs was sufficient for normalization (V2/3 < 0.01 for all PCLS). In summary, *YWHAZ* + *ACTB* seems to be a suitable potential combination of RGs for induction studies or projects studying changes in human PCLS during incubation. Despite the finding that *B2M* ranks higher among treated groups than does *ACTB*, in our liver samples, *B2M* was the least stable gene and therefore we would not recommend its use as a RG. The raw RefFinder data and results of geNorm analysis are presented in Tables S8 and S9 and Figures S4–S6.

### 3.4. Validation of Reference Genes for mRNA Normalization

The selection of RGs could have played an important role in the calculation and interpretation of our final results, especially for the time dependent findings. To show the impact of different RGs on the results of a practical experiment, the fold change of *CYP3A4* in sample L28 was analyzed (Figure 4C). Careful selection of RGs prior to our study showed that all of the tested candidate RGs were satisfactory and could be used for normalization since they are quite stable over time. Nevertheless, Figure 4 shows how much the rate and trend of target gene expression can be influenced by the RG selected in experiments using PCLS. When using *ACTB*, *B2M*, *GAPDH*, *HPRT*, or *SDHA* as a RG, the expression of *CYP3A4* increases 6–10 times within the first 18 h of incubation and then starts to decrease, while when using normalization to *YWHAZ,* the *CYP3A4* expression profile rises throughout the whole incubation period.

In order to evaluate the chosen RGs, we decided to compare the effects of the model CYP inducers BNF and RIF on the expressions of *CYP1A2* and *CYP3A4* using normalization to *YWHAZ/B2M*, respectively (Figure 4D,E). These results also enabled us to identify the interval when the target gene’s mRNA induction reached its maximum, which was found to be reached between 18 to 24 h of incubation, differing among the individual human samples. The tendency in *CYP1A2* and *CYP3A4* expression was similar in samples L28 and L37. Interestingly, there was a significant disparity between *CYP3A4* and *CYP1A2* in terms of reaching their maximum in sample L30.

### 3.5. Analysis of Expression Stability and Validation of RGs for miRNA Normalization in Human PCLS

The last part of this study focused on the selection and validation of suitable RGs for miRNA expression studies. The PCLS for miRNA expression studies were obtained from the livers of three individuals, and miRNA expression was studied at five time points within 24 h incubation. Following the information obtained from the literature, a set of five candidate miRNA RGs, namely U6, miR-16-5p, miR-23b-3p, miR-93-5p, and miR-152-3p, was tested. 

**Figure 5:**
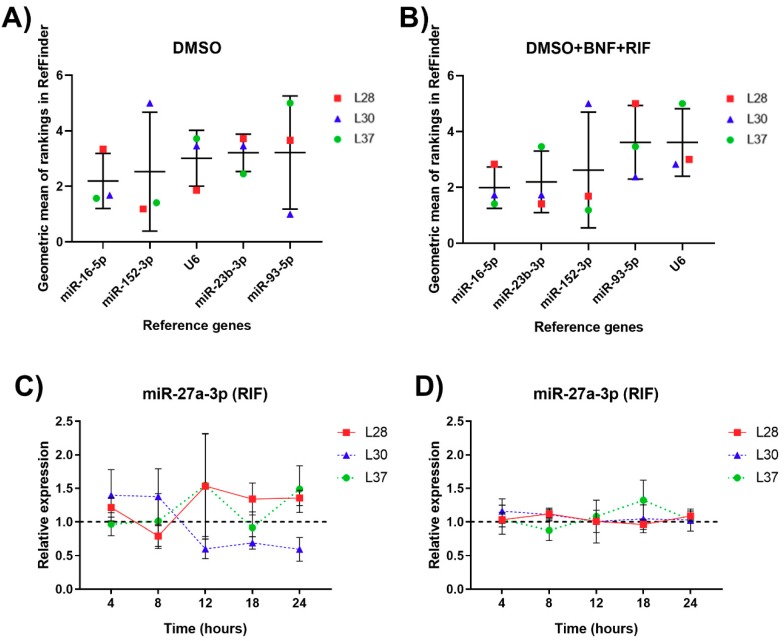
RefFinder ranking of miRNA RGs with individual values in PCLS within 24-h incubation in the control (**A**) and treated group (**B**). Relative miR-27a-3p and miR-203a-3p expression under the effect of RIF 10 µM (**C**) and BNF 10 µM (**D**), respectively, normalized to a geometric mean of miR-16-5p and miR-23b-3p. Results are presented as mean ± SD (*n* = 3).

According to the RefFinder analysis, the stability order (shown in descending stability manner) was as follows: miR-16-5p > miR-152-3p > U6 = miR-93-5p > miR-23b-3p. In the treated group, the gene stability decreased in the order miR-16-5p > miR-23b-3p > miR-152-3p > U6 = miR-93-5p (Figure 5A,B). Therefore, the most stable genes in the control group were miR-16-5p and miR-152-3p, while in the treated group, the most stable genes were miR-16-5p and miR-23b-3p. GeNorm analysis showed that a combination of two RGs was sufficient for miRNA normalization (V2/3 < 0.15 for all PCLS). Taken together, the combinations of miR-16-5p + miR-152-3p/miR-23b-3p showed promising results in the tested human samples and these combinations seem to be suitable potential RGs for induction studies or projects studying changes in human PCLS during incubation. The observed stability differences for the tested miRNAs were almost negligible. Raw RefFinder data and the results of the geNorm analysis are presented in Tables S10 and S11 and Figures S6 and S7.

The relative expression of miR-27a-3p and miR-203a-3p in the PCLS treated with RIF and BNF are presented in Figure 5C,D, respectively. Both miR-27a-3p and miR-203a-3p revealed inter-individual variability rather than the statistically significant changes caused by the inducers.

## 4. Discussion

PCLS are a model with increasing use among laboratories. Nevertheless, heterogenic handling and protocols can influence the viability and obtained results from PCLS. The issue of protocols/handling and incubation systems among laboratories was summarized by Granitzny et al. [34] and we hope that the present manuscript will provide an impetus to discuss the selection of RGs for RT-qPCR. Although the use of microarrays and next generation sequencing is becoming more widespread, these techniques have their own limitations (e.g., data normalization), and it is often recommended to validate their results through RT-qPCR. Normalization of RT-qPCR data is performed in order to eliminate sampling differences (e.g., RNA quality and quantity) as well as to observe real gene-specific variations, which should be none or minimal for a suitable internal RG. Therefore, determining suitable endogenous RGs is a crucial step in the analysis of gene expressions, as the employment of an unstable gene for data normalization could distort the obtained results and thus cause the wrong conclusions to be drawn. 

After surveying the literature regarding mRNA normalization in PCLS and not deliberately considering the differences among species, we found that various single or combined RGs have been used, for example, *ACTB* [3,7], *B2M* [3], *GAPDH* [3,7,9,10], *HPRT* [3,7,35], *RPLP0* [3], *GUSB* [35], *α-SMA* [11], and in one case, the particular RG was not mentioned [36]. Four of the most frequently used RGs (*ACTB*, *B2M*, *GAPDH*, *HPRT1*) were used in this study, and two more (*SDHA*, *YWHAZ*) were added into the tested pool of genes based on their status as RGs in liver tissue [32,33]. 

In the first phase of study, the stability of the selected RGs in thirty-five human liver tissues was verified. Based on the comprehensive ranking of individual liver samples in RefFinder, the most stably expressed RGs were *YWHAZ* and *ACTB*. According to the geNorm analysis, a combination of two RGs is sufficient for mRNA normalization in human liver samples. As shown in Figure 1D,E, the selection of RG can markedly influence the expression levels of target genes, in our case, *CYP1A2* and *CYP3A4*. 

Next, the stability order of the selected genes was checked in the larger pool of PCLS (twelve samples) only after 24 h of cultivation. In the second phase, it was verified in three liver samples that the tested RGs were capable of stable expression within 24-h cultivation in the model of PCLS. Both studies were performed under the same conditions and analyzed in the same way (controls vs. treated group). However, it is difficult to draw a definite conclusion from this experiment, as the sample size was too small. Ideally, a larger set of human samples would be necessary for a detailed study of the time profile of the candidate RGs’ expression within incubation. Unfortunately, the measurement of continuous expression is highly demanding with regard to the amount of the already extremely precious tissue.

miRNAs are short non-coding RNAs with 20–22 bases mediating post-transcriptional gene regulation. miRNAs usually bind to the 3´UTR of specific mRNAs to suppress their translation or accelerate degradation [37,38]. It is worth mentioning that a single miRNA can bind to several transcripts, and a single transcript can possess binding sites for multiple miRNAs [39]. The problem of miRNA normalization is the limited pool of RGs for many models, tissues, and body fluids. However, finding even a single stable miRNA under certain experimental conditions can be tricky, and therefore other non-coding RNAs such as small nuclear RNA U6 or small nucleolar RNAs (e.g., 204, 234, U24, or U26) are used quite often as RGs [37,38]. Nevertheless, the biochemical character of these non-coding RNAs does not reflect that of miRNAs, and the efficiency of extraction and reverse transcription might also differ from that of miRNAs [38,40,41]. In terms of miRNAs, we only found three publications regarding RT-qPCR data normalization. While two of these studies used only U6 for normalization [5,6], the third one used miR-93-5p [4] in PCLS. 

In our experiment, miR-16-5p was among the most stable tested genes, while in a previous study of ours with human PCLS treated by three different hepatotoxicants (i.e., acetaminophen, R-pulegone and R-menthofuran), miR-93-5p was more stable than U6 and miR-16-5p. Having previous experience with miR-16-5p and miR-93-5p as potential RGs, the pool of candidates was enlarged based on the study of Lamba et al., who analyzed multiple human liver samples in search of RG candidates [42]. Based on the analysis of these researchers, miR-23b and miR-152 were selected. We verified several miRNA RGs whose stability was comparable or better than U6, giving us a better chance to avoid using short non-coding RNAs that were not miRNAs for data normalization. We believe that the tested miR-16-5p, miR-23b-3p, miR-93-5p, and miR-152-3p could also be used in validations with other liver models. 

When designing an experiment, the selection of the time point of interest is very important. Unfortunately, human tissues for experimentation are scarce and difficult to obtain, and it is not always possible to perform a similar experiment with positive/negative controls and compounds of interest at multiple concentrations and at multiple time points. In optimal conditions, a single time point for the whole experiment is strongly preferred. Based on the BNF and RIF results (Figure 4), we decided that the time point of 24 h of incubation was sufficient to decide how strong a CYP inducer/tested compound is. Nevertheless, multiple timepoints would be needed to find the maximum expression if the goal is to compare inter-individual variation.

The multicellularity of PCLS in terms of gene expression represents both an advantage and risk, since this factor can influence the mRNA and miRNA expression profiles. Gene expression is regulated by different mechanisms, one them being negative regulation by microRNAs. The expression of CYPs is also partly regulated by miRNAs [43]. The expression of *CYP3A4* is directly influenced by miR-27a-3p, while miR-203a-3p indirectly regulates *CYP1A2* via the pregnane X receptor [44]. The relative expression of these miRNAs (Figure 5) shows inter-individual variability with no trend similarities and no statistically significant changes. In our search for publications describing the experimental results regarding the influence of BNF or RIF on miRNA profiles in the liver, we did not find any information about BNF, although two papers were found about the influence of 10 µM RIF on miRNA expression in primary human hepatocytes using microarrays. The results of these two articles presented a 3-fold downregulation of miR-27a after 24 h [45] and a 1.82-fold upregulation after 48 h [46], respectively. However, the miRNA expression in the first case was analyzed by differential sequencing, and in the second case by RT-qPCR normalized only to U6. On the other hand, the results from our experiments (Figure 5) displayed interindividual dependent variation rather than time- or induction-influenced variation. In the cases of *CYP3A4* and *CYP1A2*, the multicellularity did not seem to pose a problem. The multifold induction of *CYP3A4* by rifampicin and *CYP1A2* by BNF were well visible, showing interindividual differences in the rate of induction as well as in the time it took these genes to reach the peak of induction, with a peak interval of between 18 to 24 h for both genes (Figure 4).

RT-qPCR has become a routine technique, allowing the high-throughput analysis of RNA expression over a large range and at relatively low cost. Nevertheless, normalization remains a problem and it is a target of frequent criticism [47]. It would be optimal if a comparison of a selection of RGs among various laboratories and research groups could be undertaken, not to mention a comparison between the stability of RGs in human liver slices and PCLS from other species. Human tissues are especially problematic due to their variability and inter-individual differences. While *HPRT1* was the least stable in samples L28 and L30, it was the most stable gene in L38. 

## 5. Conclusions

In summary, a set of potential RGs for mRNA was tested in thirty-five human liver samples using RT-qPCR, with the combination of *YWHAZ*/*ACTB* identified in geNorm and RefFinder as the most suitable for gene normalization. Subsequently, two sets of potential RGs for mRNA and miRNA were tested in human PCLS at multiple time points during a 24 h incubation using RT-qPCR. A combination of two RGs was identified in geNorm as sufficient for gene normalization. The most stable RGs, based on the comprehensive ranking of individual liver samples in RefFinder, were determined to be *YWHAZ*/*B2M* for the control slices and *YWHAZ*/*ACTB* for the treated slices. However, the difference in the stability results for candidate RGs was small, and all of them could be used for normalization. Nevertheless, *B2M* was the least stable candidate RG in the human liver samples and therefore we would not recommend its use for data normalization in PCLS. In the case of miRNA, the most stable genes in the control and treated PCLS were miR-16-5p/miR-152-3p and miR-16-5p/miR-23b-3p, respectively, all of which were equally or more stable than the commonly used U6. The genes were found to be rather stable, especially for miRNA in the tested human PCLS. The stability of RGs must always be tested due to many variables involved, for example, variabilities in liver donor pharmacotherapy, donor diet, pathologies, and age as well as experimental conditions and handling.

## Figures and Tables

**Table 1:** Summary of human samples.

Human Sample	Gender (Age)	Reason of Surgery	Long-Term Pharmacotherapy	PCLS Preparation
L1	Female (37)	CRC ^1^	Insulin	No
L3	Male (58)	CCC ^2^	Allopurinol, felodipine, ramipril, indapamide, atorvastatin, citalopram, pregabalin	No
L4	Male (35)	Adenoma	Mesalazine, omeprazole, escitalopram, budesonide	No
L5	Male (63)	CRC	Ramipril, atorvastatin, metformin, allopurinol	Yes
L6	Male (69)	CRC	Hydrochlorothiazide	Yes
L7	Male (69)	CRC	Nitrendipine, acetylsalicylic acid	Yes
L8	Female (69)	CRC	Verapamil, trandolapril	No
L9	Male (81)	CRC	Betaxolol	Yes
L10	Female (66)	HCC ^3^	Pantoprazole	No
L11	Female (57)	CRC, liver metastases	None	Yes
L12	Female (73)	CRC	Lerkanidipine, furosemide, perindopril, nadroparin	No
L13	Female (67)	CCC	Nadroparin	No
L14	Female (45)	BFNH ^4^	None	Yes
L15	Female (69)	CRC	Nebivolol, simvastatin, digoxin, irbesartan, hydrochlorothiazide, nadroparin	No
L16	Female (59)	CRC	None	Yes
L17	Male (39)	CRC	None	No
L18	Male (83)	HCC	Lacidipine, solifenacin, tamsulosin	No
L19	Female (65)	CRC	Amlodipine	No
L20	Female (84)	Abscess	None	No
L21	Male (34)	Jejunal adenocarcinoma	None	No
L22	Female (84)	CRC	Telmisartan, nitrendipine, formoterol	No
L23	Male (83)	CRC	Furosemide, amlodipine, acetylsalicylic acid, telmisartan, salmeterol, fluticasone	No
L24	Male (77)	HCC	Metoprolol, felodipin, ramipril, metformin, gliclazid, finasteride, warfarin,nadroparin	No
L25	Male (70)	CRC	Perindopril, betaxolol, metformin, atorvastatin, allopurinol, insulin	No
L26	Male (72)	CRC	None	No
L27	Male (70)	CRC	Insulin, atorvastatin	No
L28	Female (26)	BFNH	None	Yes
L29	Male (59)	Renal cell carcinoma, liver metastases	None	No
L30	Female (81)	CRC, liver metastases	Hydrochlorothiazide, betaxolol, acetylsalicylic acid, zolpidem, insulin	Yes
L33	Female (72)	CCC	Lerkanidipine, irbesartan, hydrochlorothiazide, lansoprazole, levothyroxine, acetylsalicylic acid, fenofibrate, nebivolol	No
L34	Male (62)	CRC	Ramipril, felodipine, metoprolol, rosuvastatin, acetylsalicylic acid	No
L35	Male (72)	CRC, liver metastases	Tamsulosin, metoprolol, omeprazole	No
L36	Female (78)	CCC	Simvastatin, bisoprolol, furosemide, ramipril, enoxaparin, zolpidem	Yes
L37	Male (50)	Neuroendocrine tumor, liver metastases	Insulin	Yes
L38	Male (59)	CCC	None	Yes

^1^ CRC, colorectal carcinoma; ^2^ CCC, cholangiocellular carcinoma; ^3^ HCC, hepatocellular carcinoma; ^4^ BFNH, benign focal nodular hyperplasia.

**Table 2:** Description of selected candidate reference genes and genes of interest.

Gene Symbol	Gene Name	GeneBank or miRbase Accession Number	Gene Function	Primer Sequences 5´-3´	Tm^1^ (°C)	E^2^ (%)
**Candidate reference genes for mRNA normalization**						
*ACTB*	Actin beta	NM_001101.4	Structural protein of cytoskeleton	F^3^: TCCCTGGAGAAGAGCTACGAG R^4^: CAGGAAGGAAGGCTGGAAGAG	86.5	102
*B2M*	Beta-2-microglobulin	NM_004048.2	Beta-chain of major histocompatibility complex	F: TGCTGTCTCCATGTTTGATGTATC R: TCTCTGCTCCCCACCTCTAAG	83	99
*GAPDH*	Glyceraldehyde-3-phosphate dehydrogenase	NM_002046	Enzyme of glycolysis pathway	F: GAGTCCACTGGCGTCTTCAC R: GAGGCATTGCTGATGATCTTGAG	86	101
*HPRT1*	Hypoxanthine phosphoribosyltransferase 1	NM_000194.2	Metabolism of purines	F: TGGTCAGGCAGTATAATCCAAAGA R: TTCAAATCCAACAAAGTCTGGCT	82	101
*SDHA*	Succinate dehydrogenase complex, subunit A	NM_004168.3	Critical function in mitochondrial respiratory chain	F: TGGGAACAAGAGGGCATCTG R: ACCACCACTGCATCAAATTCATG	79.5	99
*YWHAZ*	Tyrosine 3-monooxygenase/tryptophan 5-monooxygenase activation protein zeta	NM_003406.3	Important protein for many signal transduction pathways	F: TGATCCCCAATGCTTCACAAG R: GCCAAGTAACGGTAGTAATCTCC	77.5	102
**Candidate reference genes for miRNA normalization**						
miR-16-5p	MicroRNA 16 (5p)	MIMAT0000069	Regulation of apoptosis	RT^5^: GTCTCCTCTGGTGCAGGGTCCGAG GTATTCGCACCAGAGGAGACCGCC AA F: ACAGCCTAGCAGCACGTAAAT	79	102
miR-23b-3p	MicroRNA 23b (3p)	MIMAT0000418	Associated with cell proliferation, invasion, and apoptosis	RT: GTCTCCTCTGGTGCAGGGTCCGAGGTA TTCGCACCAGAGGAGACGTGGTA F: ATCTGTATCACATTGCCAGGGA	77.5	109
miR-93-5p	MicroRNA 93 (5p)	MIMAT0000093	OncomiR, plays an essential role in tumorigenesis and progression of various carcinomas	RT: GTCTCCTCTGGTGCAGGGTCCGAGGTATTCGCACCAGAGGAGAC F: GTCAATCAAAGTGCTGTTCGTG	78.5	105
miR-152-3p	MicroRNA 152 (3p)	MIMAT0000438	Regulates hepatic glycogenesis, tumor suppressor	RT: GTCTCCTCTGGTGCAGGGTCCGAGGTA TTCGCACCAGAGGAGACCCAAGT F: CGACGTTCAGTGCATGACAG	78.5	100
U6	Small nuclear RNA U6	NR_003027	RNA splicing	R: AACGCTTCACGAATTTGCGTG F: GCTCGCTTCGGCAGCACA	80.5	99
universal				R: GAGGTATTCGCACCAGAGGA		
**Genes of interest**						
CYP1A2	Cytochrome P450 family 1 subfamily A member 2	NM_000761	Phase I biotransformation	F: CTTCCCTGAGAGTAGCGATGAGA R: GCAGTCTCCACGAACTCATGAG	85.5	101
CYP3A4	Cytochrome P450 family 3 subfamily A member 4	NM_017460.5	Phase I biotransformation	F: CCCCTGAAATTAAGCTTAGGAGG R: CTGGTGTTCTCAGGCACAGA	82.5	99
miR-27a-3p	MicroRNA 27a (3p)	MIMAT0000084	Direct regulation of CYP3A4	RT: GTCTCCTCTGGTGCAGGGTCCGAGGTA TTCGCACCAGAGGAGACGCGGAA F: CGGCGTTTCACAGTGGCTAA	80.5	106
miR-203a-3p	MicroRNA 203a (3p)	MIMAT0000264	Indirect regulation of CYP1A2 via PXR receptor	RT: GTCTCCTCTGGTGCAGGGTCCGAGGTA TTCGCACCAGAGGAGACCTAGTG F: CGGCGTGTGAAATGTTTAGGA	78.5	105

^1^ Tm, melting temperature; ^2^ E, assays efficiency; ^3^ F, forward primer; ^4^ R, reverse primer; ^5^ RT, reverse transcription primer.

## Supplementary Materials

The following are available online at https://www.mdpi.com/2073-4425/10/10/763/s1, Figure S1: Melting curve plots; Figure S2: Determination of the optimal number of RGs in control PCLS by geNorm analysis; Figure S3: Determination of the optimal number of RGs in treated PCLS by geNorm analysis; Figure S4: Determination of the optimal number of RGs in control PCLS by geNorm analysis; Figure S5: Determination of the optimal number of RGs in treated PCLS by geNorm analysis; Figure S6: Determination of the optimal number of RGs in the control PCLS by geNorm analysis; Figure S7: Determination of the optimal number of RGs in treated PCLS by geNorm analysis; Table S1: MIQE checklist; Table S2: Summary of RIN values; Table S3: Summary of liver biochemical parameters; Table S4: RefFinder results for mRNA of human liver tissues (n = 35); Table S5: Effects of different normalization approaches on the expression of CYP1A2 and CYP3A4. Results are presented as 2-ΔΔCq values relative to sample L1.; Table S6: RefFinder results for the mRNA of the control PCLS incubated for 24 h with DMSO; Table S7: RefFinder results for the mRNA of the treated PCLS incubated for 24 h with RIF/BNF/DMSO; Table S8: RefFinder results for the mRNA of the control PCLS incubated for 24 h with DMSO (time dependence); Table S9: RefFinder results for the mRNA of the treated PCLS incubated for 24 h with RIF/BNF/DMSO (time dependence); Table S10: RefFinder results for the miRNA of the control PCLS incubated for 24 h with DMSO (time dependence); Table S11: RefFinder results for the miRNA of the treated PCLS incubated for 24 h with RIF/BNF/DMSO (time dependence).
