# Supplementary material for: The Selection and Validation of Reference Genes for mRNA and microRNA Expression Studies in Human Liver Slices Using RT-qPCR
Source: Genes (Basel). 2019 Sep 28;10(10):763. doi: 10.3390/genes10100763 (PMC6826422; doi:10.3390/genes10100763)
Supplement: Supplementary file 1 [file genes-10-00763-s001.pdf]

The Selection and Validation of Reference Genes for mRNA and microRNA Expression Studies in Human Liver Slices Using RT-qPCR

Tomáš Zárybnický, Petra Matoušková, Martin Ambrož, Zdeněk Šubrt, Lenka Skálová and Iva Boušová

Table S1. Selection of RIN values in human liver and human PCLS.

**Human liver**		**Human PCLS**	
**Sample**	**RIN value**	**Sample**	**RIN value**
L1	6,1	L28 C4	6,3
L7	7,3	L28 BNF8	6,3
L10	6,9	L28 RIF24	6,9
L17	6,9	L30 RIF18	6,8
L19	6,7	L30 BNF4	6,9
L22	6,8	L30 RIF24	6,4
L28	7,0	L38 C4	8,7
L36	6,1	L38 BNF8	8,2
L38	6,7	L38 C24	7,8

Table S2. MIQE checklist.

ITEM TO CHECK	IMPORTANCE	CHECKLIST
Definition of experimental and control groups	**E**	Precision-cut human liver slices (8 mm diameter, 150 μm thickness) treated by DMSO (control group) or by model cytochrome P450 inducers β-naphthoflavone and rifampicin (treated group).
Number within each group	**E**	Three human samples, five time points for each sample, triplicates for every treatment.
Assay carried out by core lab or investigator's lab?	D	Investigator´s laboratory
Acknowledgement of authors' contributions	D	Conceived and designed the experiments: TZ PM BS. Performed the experiments: TZ MA. Analyzed the data: TZ IB. Contributed reagents/materials/analysis tools: ZS IB. Wrote the paper: TZ IB LS. All authors read and approved the final manuscript.
Description	**E**	Fresh human liver from partial hepatectomy.
Volume/mass of sample processed	D	8 mm diameter, 150 μm thickness
Microdissection or macrodissection	**E**	Macrodissection
Processing procedure	**E**	Piece of human liver was divided into 1 cm thick slices, cylinders of 8 mm diameter were punched out of the tissue with hollow bits, the cylinders were further sliced with Krumdieck tissue slicer MD4000 into desired thickness.
If frozen - how and how quickly?	**E**	Liver slices were frozen in powdered dry ice and further stored in freezer at -80°C
If fixed - with what, how quickly?	**E**	Not fixed
Sample storage conditions and duration (especially for FFPE samples)	**E**	Samples were held at -80°C at most up to two months before RNA isolation
Procedure and/or instrumentation	**E**	Homogenization of samples was performed using a single metal bead in 2 ml Eppendorf tube using 0.5 ml of TriReagent per single slice of liver tissue. Total RNA was extracted using the TriReagent (Biotech) following manufacture's protocol. The purified RNA was dissolved in 40 μl DEPC-Treated Water (0.01% DEPC in HPLC water, autoclaved) and stored at -80°C
Name of kit and details of any modifications	**E**	TriReagent (Biotech, TR-118). We exactly followed manufacture's protocol.
Source of additional reagents used	D	Chloroform (Chemapol); 2-propanol (Sigma-Aldrich); absolute Ethanol (Sigma-Aldrich); DEPC (Sigma-Aldrich)
Details of DNase or RNAse treatment	**E**	Four μg of RNA were treated with 2 U of DNase I (Biotech) in 30 μl final volume reaction. Digestion of DNA was achieved with 30 minutes incubation at 37°C. Then 1.5 μl of 100 mM EDTA was added and the reaction was incubated for 10 min at 65°C for inactivation of DNase and diluted up to 40 μl with DEPC-treated water.
Contamination assessment (DNA or RNA)	**E**	no RT controls were performed in initial experiments with liver samples only
Nucleic acid quantification	**E**	RNA concentration was determined by measuring the absorbance at 260 nm UV light
Instrument and method	**E**	NanoDrop ND-1000 UV-Vis Spectrophotometer (Thermo Scientific)
Purity (A260/A280)	D	The absorbance ratio 260/280 of all samples proceed was higher than 1.9
Yield	D	-
RNA integrity method/instrument	**E**	RNA integrity was assessed using Agilent 2000 Bioanalyzer
RIN/RQI or Cq of 3' and 5' transcripts	**E**	Only samples with RIN>6 were used.
Electrophoresis traces	D	-

Inhibition testing (Cq dilutions, spike or other)	**E**	The standard curve has been considered sufficient to rule out the presence of inhibitors of reverse-transcription activity or PCR, also considering the high quality of starting RNAs
Complete reaction conditions	**E**	REVERSE TRANSCRIPTION OF mRNA: 5 µl of RNA (from the DNase I treatment reaction mixture = 1 µg of RNA) was firstly incubated with 1 µl random hexamers 0.1 mM. This mixture was heated to 65°C for 5 min and then incubated on ice for at least 1 min for primer annealing and spin down shortly. Finally, the sample was mixed with 4 µl 5X ProtoScript II RT Reaction Buffer, 2 µl 10x DTT, 2 µl dNTP Mix 5 mM, 3.5 µl H_2O and 0.5 µl ProtoScript II 200 U/µl was added and mixed by pipetting. Reactions were incubated in a PCR MJ Mini (Bio-Rad) at 25°C for 10 min, 42°C for 50 min and 80°C for 5 min to stop the reaction.<br>REVERSE TRANSCRIPTION OF microRNA: 2.5 µl of RNA (from the DNase I treatment reaction mixture = 250 ng of RNA) was firstly incubated with 2 µl of multiplex of stem-loop oligonucleotides specific for each miRNA and specific primer for U6 (each primer 0.71 µM). This mixture was heated to 65°C for 5 min and then incubated on ice for at least 1 min for primer annealing and spin down shortly. Finally, the sample was mixed with 2 µl 5x ProtoScript II RT Reaction Buffer, 1 µl 10x DTT, 2 µl dNTP Mix 5 mM, 0.2 µl RNase Inhibitor 40 U/µl and 0.3 µl ProtoScript II 200 U/µl and mixed by pipetting. Reactions were incubated in a PCR MJ Mini (Bio-Rad) at 16°C for 30 min, 42°C for 50 min and 95°C for 5 min to stop the reaction.
Amount of RNA and reaction volume	**E**	Amount of RNA for mRNA: 1 µg; Reaction volume: 20 µl; for miRNA: 0.25 µg; Reaction volume 10 µl
Priming oligonucleotide (if using GSP) and concentration	**E**	random hexamers: 100 µM; gene specific primers and stem loop-oligos for microRNAs: 5 µM
Reverse transcriptase and concentration	**E**	ProtoScript II (NEB) at final concentration 10 U/µl
Temperature and time	**E**	mRNA: 65°C for 5 min, 25°C for 10 min, 42°C for 50 min and 80°C for 5 min<br>microRNA: 65°C for 5 min, 16°C for 30 min, 42°C for 30 min and 95°C for 5 min
Manufacturer of reagents and catalogue numbers	D	DNase I (Biotech, M0303S); dNTP Mix with dTTP (Eurogentec, NU-0010-10); ProtoScript II (NEB, Cat. M0368L); RNase Inhibitor (Biotech, M0307S)
Cqs with and without RT	D*	-
Storage conditions of cDNA	D	-20°C
If multiplex, efficiency and LOD of each assay.	**E**	-
Sequence accession number	**E**	Table 2
Location of amplicon	D	-
Amplicon length	**E**	B2M-86nt; ACTB-99nt; GAPDH-164nt; HPRT1-101nt; SDHA-87nt; YWHAZ-76nt
In silico specificity screen (BLAST, etc)	**E**	Specificity tested by BLAST (NCBI); all primer pairs were specific except ACTB, which could amplify longer fragment of other gene (>1000nt) which is unlikely due to short synthesis step in qPCR, gene-specific amplification was confirmed by a single peak in melting curve analysis.
Pseudogenes, retropseudogenes or other homologs?	D	-
Sequence alignment	D	-
Secondary structure analysis of amplicon	D	-
Location of each primer by exon or intron (if applicable)	**E**	B2M(forward:4th exon,reverse:4th exon); ACTB(f:4th,r:4th); GAPDH(f:5th,r:6/7th); HPRT1(f:6th, r:7/8th); SDHA (f:2nd,r:3rd); YWHAZ (f:3rd,r:3rd)
What splice variants are targeted?	**E**	-
Primer sequences	**E**	Table 2
RTPrimerDB Identification Number	D	Table 2
Probe sequences	D**	n/a

Location and identity of any modifications	**E**	no modifications were done
Manufacturer of oligonucleotides	D	Generi Biotech, Hradec Králové, Czech Republic
Purification method	D	desalted, microRNA RT primers OPC purified
Complete reaction conditions	**E**	PCR reactions were performed using QuantStudio 6 Flex (Applied Biosystems, Foster City, CA, USA) using SYBR Green I at final volume of 20 µl. Reaction mix consisted of 10 µl Xceed qPCR SG 2x Mix (Institute of Applied Biotechnologies, Prague, Czech Republic), 4.2 µl of H_2O 0.4 µl of each primer (5 µM forward and reverse primers) and 5 µl of diluted cDNA. The PCR reactions were initiated with 10-minute incubation at 95°C, followed by 40 cycles of 95°C for 10 seconds, 60°C for 40 seconds. All reactions were performed in duplicate.
Reaction volume and amount of cDNA/DNA	**E**	Reaction volume: 20 µl; amount of cDNA: 5 µl for mRNA and microRNA
Primer, (probe), Mg++ and dNTP concentrations	**E**	each primer:100nM; others not known-commercial premix (Xceed qPCR SG 2x Mix Institute of Applied Biotechnologies, Prague, Czech Republic)
Polymerase identity and concentration	**E**	not known-commercial premix (Xceed qPCR SG 2x Mix Institute of Applied Biotechnologies, Prague, Czech Republic)
Buffer/kit identity and manufacturer	**E**	Xceed qPCR SG Mix (Institute of Applied Biotechnologies, Prague, Czech Republic)
Exact chemical constitution of the buffer	D	-
Additives (SYBR Green I, DMSO, etc.)	**E**	SYBR Green I diluted in DMSO, both components of the qPCR kit
Manufacturer of plates/tubes and catalogue number	D	96 well plates (SSIbio 3425-00) sealed with MicroAmp Optical Adhesive Film (Applied Biosystems)
Complete thermocycling parameters	**E**	Initial denaturation: 95°C for 10 minutes, then 40 cycles at 95°C for 10 seconds, 60°C for 40 seconds
Reaction setup (manual/robotic)	D	manual
Manufacturer of qPCR instrument	**E**	Applied Biosystems
Evidence of optimisation (from gradients)	D	-
Specificity (gel, sequence, melt, or digest)	**E**	Melting curve analysis, ramping from 55°C to 95°C, in 0.5°C steps where fluorescence data are measured every 30 s (measured melting temperature values are provided in Table 2, curves in Figure S8)
For SYBR Green I, Cq of the NTC	**E**	The signal of the amplification plot was late (Ct > 34). The difference between the negative control and all the cDNA sample was > 9 cycles. For most of the microRNA in the NTC samples was not detected any Ct or the difference between samples and negative control was > 9 cycles.
Standard curves with slope and y-intercept	**E**	ACTB: y = -3.2818x + 28.864, B2M: y = -3.3456x + 35.296, GAPDH: y = -3.2886x + 28.382, HPRT1: y = -3.3299x + 36.085, SDHA: y = -3.3353x + 37.744, YWHAZ: y = -3.2635x + 33.259<br>miR-16: y = -3.2705x + 35.053, miR-23b: y = -3.1101x + 34.607, miR-27a: y = -3.1036x + 40.262, miR-93: y = -3.181x + 40.387, miR-152: y = -3.2226x + 38.112, miR-203: y = -3.4306x + 40.171, U6: y = 3.301x + 27.541
PCR efficiency calculated from slope	**E**	ACTB: 102%, B2M: 99%, GAPDH: 101%, HPRT1: 101%, SDHA: 99%, YWHAZ: 102%<br>miR-16: 102%, miR-23b: 109%, miR-27a: 106%, miR-152: 104%, miR-203: 93%, U6: 101%
Confidence interval for PCR efficiency or standard error	D	-
r2 of standard curve	**E**	ACTB: 0.9998, B2M: 0.9923, GAPDH: 0.9963, HPRT1: 0.9979, SDHA: 0.9998, YWHAZ: 0.9974<br>miR-16: 0.9991, miR-23b: 0.9964, miR-27a: 0.9999, miR-152: 0.9967, miR-203: 0.9955, U6: 0.9985
Linear dynamic range	**E**	The linear dynamic range was considered taking into account the linearity of the standard curves; For mRNA and microRNA: from 1/5 dilution of cDNA to 1/625 dilution;
Cq variation at lower limit	**E**	not detected
Confidence intervals throughout range	D	-

Evidence for limit of detection	**E**	not detected, the dilutions of cDNA performed in linear range defined by standard curve
If multiplex, efficiency and LOD of each assay.	**E**	-
qPCR analysis program (source, version)	**E**	QuantStudio 6 and 7 Flex Software
Cq method determination	**E**	The threshold is set manually to the level where the fluorescence rises above detection limit. The threshold specifies Ct values.
Outlier identification and disposition	**E**	None of the Cq values was discarded
Results of NTCs	**E**	The signal of the amplification plot was late (Ct > 34). The difference between the negative control and all the cDNA sample was > 9 cycles. For most of the microRNA in the NTC samples was not detected any Ct or the difference between samples and negative control was > 9 cycles
Justification of number and choice of reference genes	**E**	This is a study for the selection of reference genes
Description of normalisation method	**E**	Described in text
Number and concordance of biological replicates	D	Three biological replicates
Number and stage (RT or qPCR) of technical replicates	**E**	qPCR reactions were performed in duplicate
Repeatability (intra-assay variation)	E	Mean standard deviation of duplicates: 0.15
Reproducibility (inter-assay variation, %CV)	D	-
Power analysis	D	-
Statistical methods for result significance	**E**	two-way ANOVA with multiple comparisons and Sidak *post hoc* test, scored as significant when $p<0.005$
Software (source, version)	E	GraphPad Prism 8
Cq or raw data submission using RDML	**D**	-

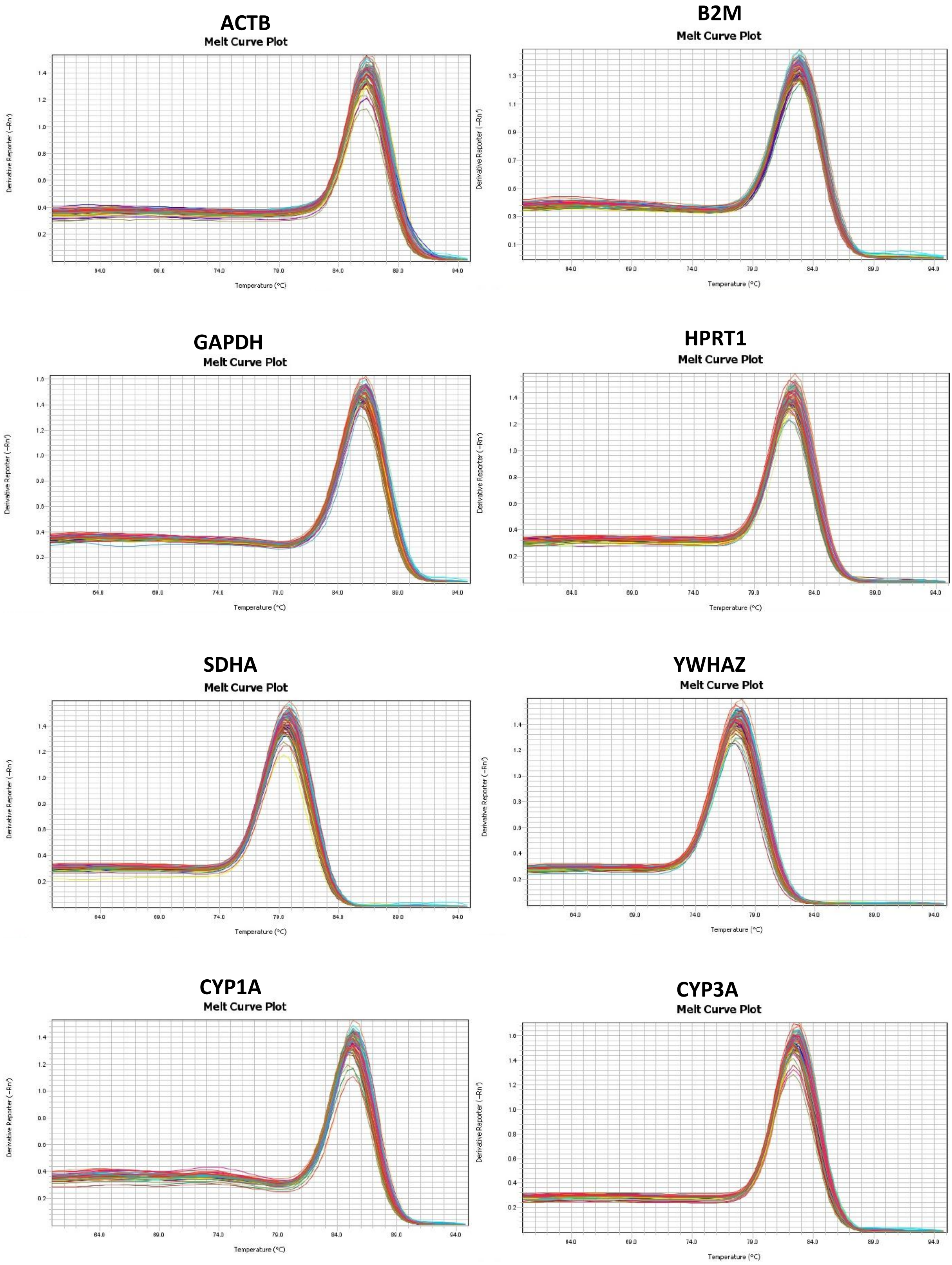
ACTB
Melt Curve Plot
Derivative Reporter (−Rn')
Temperature (°C)
B2M
Melt Curve Plot
Derivative Reporter (−Rn')
Temperature (°C)
GAPDH
Melt Curve Plot
Derivative Reporter (−Rn')
Temperature (°C)
HPRT1
Melt Curve Plot
Derivative Reporter (−Rn')
Temperature (°C)
SDHA
Melt Curve Plot
Derivative Reporter (−Rn')
Temperature (°C)
YWHAZ
Melt Curve Plot
Derivative Reporter (−Rn')
Temperature (°C)
CYP1A
Melt Curve Plot
Derivative Reporter (−Rn')
Temperature (°C)
CYP3A
Melt Curve Plot
Derivative Reporter (−Rn')
Temperature (°C)

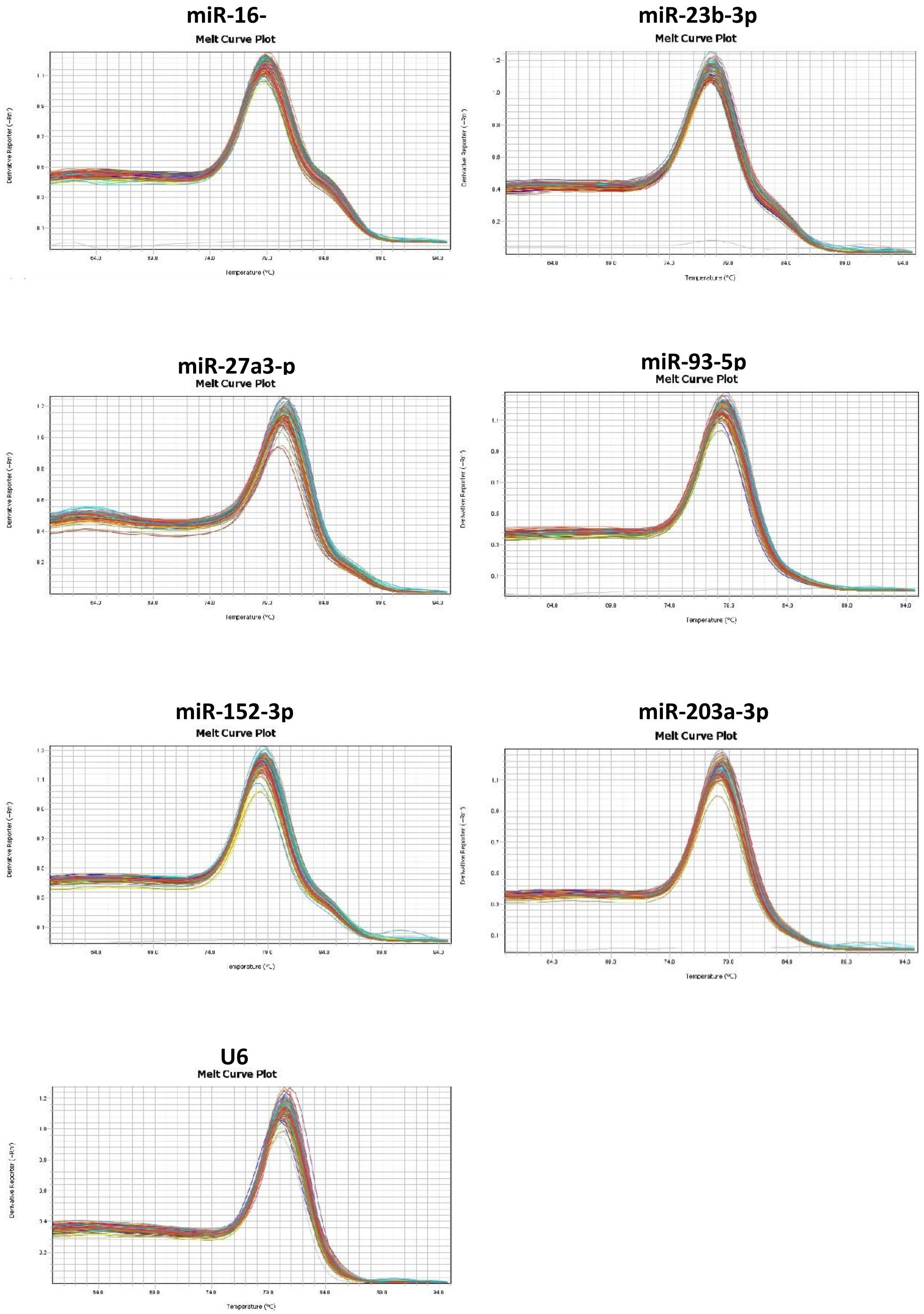

Figure S1. Melting curve plots.

Table S3. Summary of liver biochemical parameters.

Human sample	Gender (age)	Reason of surgery	Total BIL (μmol/L)	ALT (μkat/L)	AST (μkat/L)	GMT (μkat/L)	ALP (μkat/L)
L1	Female (73)	Colorectal carcinoma	5.5	0.35	0.38	**1.78**	1.99
L3	Male (58)	Cholangiocellular carcinoma	22.0	0.50	0.37	0.64	1.24
L4	Male (35)	Adenoma	10.0	0.25	0.28	0.80	1.44
L5	Male (63)	Colorectal carcinoma	11.5	0.55	0.44	**0.88**	1.60
L6	Male (69)	Colorectal carcinoma	**48.9**	0.34	0.45	**1.10**	0.98
L7	Male (69)	Colorectal carcinoma	7.0	0.43	0.46	**1.32**	0.69
L8	Female (69)	Colorectal carcinoma	10.8	0.42	0.39	0.98	0.93
L9	Male (81)	Colorectal carcinoma	10.8	0.42	0.39	**0.93**	0.98
L10	Female (66)	Hepatocellular carcinoma	12.4	0.33	0.47	**1.15**	1.49
L11	Female (57)	Liver metastasis from colorectal carcinoma	5.0	0.35	0.39	**1.97**	0.61
L12	Female (73)	Colorectal carcinoma	6.0	0.31	0.40	**1.14**	0.73
L13	Female (67)	Cholangiocellular carcinoma	7.0	0.32	0.35	**3.50**	**1.89**
L14	Female (45)	Benign focal nodular hyperplasia	7.0	0.47	0.47	0.35	0.84
L15	Female (69)	Colorectal carcinoma	21.2	0.41	0.36	0.88	0.75
L16	Female (59)	Colorectal carcinoma	9.4	0.29	0.45	0.51	**1.80**
L17	Male (39)	Colorectal carcinoma	12.6	0.26	0.33	0.25	0.98
L18	Male (83)	Hepatocellular carcinoma	14.0	0.34	0.57	1.32	**2.49**
L19	Female (65)	Colorectal carcinoma	9.7	0.51	0.41	**1.13**	1.20
L20	Female (84)	Abscess	5.0	0.44	0.28	0.73	1.03
L21	Male (34)	Jejunal adenocarcinoma	6.0	**2.24**	**1.38**	**3.42**	**4.93**
L22	Female (84)	Colorectal carcinoma	17.2	0.21	0.33	0.44	2.30
L23	Male (83)	Colorectal carcinoma	**39.0**	0.32	0.37	0.72	ND
L24	Male (77)	Hepatocellular carcinoma	20.1	0.51	0.43	1.01	1.59
L25	Male (70)	Colorectal carcinoma	18.1	0.71	0.53	**1.31**	1.27

L26	Male (72)	Colorectal carcinoma	7.0	0.34	0.38	1.14	**2.33**
L27	Male (70)	Colorectal carcinoma	11.2	0.64	0.49	**1.71**	2.16
L28	Female (26)	Benign focal nodular hyperplasia	15.1	0.40	0.55	**1.23**	1.28
L29	Male (59)	Liver metastases from renal cell carcinoma	6.0	0.36	0.30	**1.37**	**2.34**
L30	Female (81)	Liver metastasis from colorectal carcinoma	4.0	0.25	0.20	0.42	1.42
L33	Female (72)	Cholangiocellular carcinoma	11.3	0.58	**0.74**	**2.34**	0.86
L34	Male (62)	Colorectal carcinoma	13.7	0.61	0.61	**3.48**	**2.24**
L35	Male (72)	Liver metastasis from colorectal carcinoma	10.2	0.41	0.63	0.39	1.16
L36	Female (78)	Cholangiocellular carcinoma	14.2	0.50	0.45	1.26	1.40
L37	Male (50)	Metastasis from neuroendocrine tumour	13.5	**1.55**	**0.91**	**4.72**	**2.87**
L38	Male (59)	Cholangiocellular carcinoma	20.2	0.33	0.32	1.21	0.61

Reference range for individual analytes: Total bilirubin (BIL): male 0–25 µmol/L, female 0–17 µmol/L. Alanine aminotransferase (ALT): male 0.17-0.83 µkat/L, female 0.17–0.58 µkat/L. Aspartate aminotransferase (AST): male 0.17–0.85 µkat/L, female 0.17–0.60 µkat/L. γ-Glutamyl transferase (GMT): male 0–1.00 µkat/L, female 0–0.67 µkat/L. Alkaline phosphatase (ALP): male 0.66-2.20 µkat/L, female 0.58–2.35 µkat/L. Values marked in bold red letters exceeded upper reference limit.

Table S4. RefFinder results for mRNA of human liver tissues (n=35).

**mRNA stability in human liver tissue**												
**Comprehensive ranking**			**Delta CT**		**BestKeeper**			**NormFinder**		**geNorm**		
Rank	Genes	Geomean of ranking values	Rank	Average of SD	Rank	SD	Pearson correlation coefficient (*r*)	Rank	Stability value	Rank	Stability value	
2	ACTB	2.632	4	1.030	3	1.002	0.874	4	0.725	1	0.742	
6	B2M	4.729	5	1.106	4	1.113	0.820	5	0.835	4	0.973	
3	GAPDH	2.991	2	0.998	5	1.175	0.941	2	0.661	3	0.884	
5	HPRT1	3.834	6	1.198	1	0.376	0.663	6	0.982	5	1.048	
4	SDHA	3.568	3	1.026	6	1.219	0.941	3	0.716	2	0.869	
1	YWHAZ	1.189	1	0.931	2	0.921	0.911	1	0.502	1	0.742	

Table S5. Effects of different normalisation approaches on the expression of *CYP1A2* and *CYP3A4*. Results are presented as $2^{-\Delta\Delta Cq}$ values relative to sample L1.

Liver No.	*CYP1A2*					*CYP3A4*				
	YWHAZ	*ACTB*	*YWHAZ:ACTB*	*B2M*	*B2M:HPRT1*	*YWHAZ*	*ACTB*	*YWHAZ:ACTB*	*B2M*	*B2M:HPRT1*
L1	1.00	1.00	1.00	1.00	1.00	1.00	1.00	1.00	1.00	1.00
L3	0.97	1.74	1.34	1.77	2.12	0.49	0.87	0.67	0.88	1.06
L4	1.12	2.09	1.59	1.44	1.54	0.61	1.14	0.87	0.78	0.84
L5	0.17	2.46	0.72	0.19	0.20	0.41	6.14	1.80	0.48	0.51
L6	0.65	0.87	0.77	0.49	0.40	1.30	1.75	1.54	0.99	0.80
L7	0.35	0.60	0.47	0.64	0.81	0.21	0.36	0.28	0.38	0.49
L8	0.46	0.47	0.47	0.64	0.42	0.53	0.54	0.54	0.74	0.48
L9	0.17	0.23	0.20	0.24	0.14	0.10	0.14	0.12	0.14	0.08
L10	0.92	1.14	1.04	0.91	1.04	0.86	1.07	0.98	0.85	0.97
L11	0.90	1.61	1.27	0.49	0.27	0.41	0.74	0.58	0.23	0.12
L12	0.20	0.22	0.21	0.23	0.23	0.41	0.45	0.43	0.46	0.47
L13	0.13	0.24	0.19	0.23	0.20	0.11	0.21	0.16	0.19	0.17
L14	0.65	1.09	0.87	1.27	0.90	1.09	1.85	1.47	2.14	1.52
L15	0.10	0.15	0.13	0.08	0.12	0.16	0.24	0.20	0.12	0.19
L16	0.30	0.51	0.40	0.64	0.57	0.18	0.30	0.24	0.37	0.34
L17	1.07	1.22	1.15	3.60	2.79	0.86	0.98	0.92	2.88	2.24
L18	0.12	0.14	0.13	0.13	0.14	0.46	0.55	0.51	0.51	0.54
L19	0.86	0.94	0.90	3.55	2.48	1.29	1.41	1.35	5.32	3.71
L20	0.25	0.58	0.40	0.33	0.37	0.18	0.41	0.29	0.24	0.26
L21	0.10	0.26	0.17	0.36	0.36	0.04	0.11	0.07	0.15	0.15
L22	0.22	0.67	0.41	0.25	0.28	0.37	1.13	0.69	0.42	0.47
L23	0.48	0.65	0.57	0.64	0.49	1.19	1.61	1.42	1.59	1.22
L24	0.13	0.32	0.21	0.80	0.49	0.10	0.25	0.17	0.63	0.39
L25	0.81	0.80	0.82	1.78	0.93	0.65	0.64	0.65	1.42	0.75
L26	0.60	0.71	0.66	1.92	1.73	0.26	0.31	0.29	0.83	0.76
L27	0.80	1.37	1.08	1.36	1.38	0.31	0.53	0.42	0.53	0.53
L28	0.75	1.20	0.98	6.21	2.67	0.39	0.63	0.51	3.26	1.40
L29	0.17	0.19	0.19	0.31	0.19	0.40	0.44	0.42	0.71	0.44
L30	0.07	0.21	0.13	0.31	0.10	0.12	0.36	0.22	0.53	0.16
L33	0.35	0.33	0.35	0.40	0.21	0.71	0.67	0.70	0.80	0.43
L34	0.06	0.13	0.09	0.26	0.18	0.05	0.11	0.08	0.22	0.16
L35	1.03	1.15	1.09	4.67	2.86	0.73	0.82	0.78	3.33	2.04
L36	0.92	0.75	0.82	6.15	2.91	0.96	0.79	0.86	6.46	3.06
L37	0.91	1.06	1.00	2.81	1.88	0.59	0.69	0.64	1.82	1.21
L38	0.39	0.62	0.51	3.21	2.02	0.14	0.22	0.18	1.12	0.71

Table S6. RefFinder results for mRNA of control PCLS incubated for 24 h with DMSO.

Sample No.	**mRNA stability in human liver slices treated by DMSO**											
	**Comprehensive ranking**			**Delta CT**		**BestKeeper**			**NormFinder**		**geNorm**	
	Rank	Genes	Geomean of ranking values	Rank	Average of SD	Rank	SD	Pearson correlation coefficient (*r*)	Rank	Stability value	Rank	Stability value
L5	3	ACTB	2.630	3	0.37	2	0.20	0.929	1	0.150	3	0.252
	1	B2M	1.73	1	0.32	3	0.33	0.996	2	0.095	2	0.173
	6	GAPDH	5.73	6	0.66	5	0.51	0.662	6	0.609	5	0.436
	2	HPRT1	2.21	2	0.36	4	0.47	0.981	3	0.207	1	0.070
	5	SDHA	3.34	5	0.51	1	0.17	0.317	5	0.448	4	0.327

	4	YWHAZ	3.13	4	0.41	6	0.53	0.981	4	0.307	1	0.070
L6	Rank	Genes	Geomean of ranking values	Rank	Average of SD	Rank	SD	Pearson correlation coefficient (*r*)	Rank	Stability value	Rank	Stability value
	2	ACTB	2.06	3	0.334	2	0.53	0.988	3	0.079	1	0.029
	4	B2M	3.344	5	0.612	1	0.53	0.805	5	0.563	4	0.373
	6	GAPDH	5.733	6	0.623	5	0.81	0.948	6	0.580	5	0.456
	5	HPRT1	4.427	4	0.508	6	0.82	0.962	4	0.370	3	0.275
	3	SDHA	2.449	2	0.332	3	0.54	1.000	2	0.051	2	0.080
	1	YWHAZ	1.414	1	0.329	4	0.56	0.989	1	0.014	1	0.029
L7	Rank	Genes	Geomean of ranking values	Rank	Average of SD	Rank	SD	Pearson correlation coefficient (r)	Rank	Stability value	Rank	Stability value
	1	ACTB	1.682	1	0.135	4	0.12	0.923	2	0.088	1	0.011
	5	B2M	4.949	5	0.176	6	0.13	0.816	5	0.124	3	0.097
	2	GAPDH	1.861	2	0.139	2	0.09	0.819	1	0.077	2	0.031
	3	HPRT1	2.783	3	0.142	5	0.12	0.920	4	0.105	1	0.011
	5	SDHA	5.045	6	0.267	3	0.11	0.001	6	0.252	5	0.171
	4	YWHAZ	2.783	4	0.166	1	0.06	0.346	3	0.089	4	0.123
L9	Rank	Genes	Geomean of ranking values	Rank	Average of SD	Rank	SD	Pearson correlation coefficient (*r*)	Rank	Stability value	Rank	Stability value
	5	ACTB	5.233	5	0.281	6	0.22	0.980	5	0.262	4	0.183
	4	B2M	2.913	3	0.197	4	0.11	0.950	2	0.059	2	0.126
	1	GAPDH	1.189	1	0.190	2	0.07	0.546	1	0.030	1	0.100
	2	HPRT1	2.060	2	0.194	3	0.11	0.900	3	0.097	1	0.100
	5	SDHA	5.733	6	0.347	5	0.17	0.001	6	0.335	5	0.236
	3	YWHAZ	2.828	4	0.27	1	0.04	0.001	3	0.115	3	0.155
L11	Rank	Genes	Geomean of ranking values	Rank	Average of SD	Rank	SD	Pearson correlation coefficient (*r*)	Rank	Stability value	Rank	Stability value
	3	ACTB	2.828	2	0.262	4	1.20	0.997	2	0.108	3	0.168
	4	B2M	3.310	4	0.315	2	1.02	0.999	3	0.176	4	0.226
	2	GAPDH	2.763	3	0.273	5	1.28	1.000	4	0.182	1	0.111
	5	HPRT1	3.500	5	0.322	6	1.33	0.999	5	0.285	1	0.111
	6	SDHA	3.834	6	0.513	1	0.84	0.985	6	0.498	5	0.321
	1	YWHAZ	1.732	1	0.244	3	1.17	0.999	1	0.067	2	0.158
L14	Rank	Genes	Geomean of ranking values	Rank	Average of SD	Rank	SD	Pearson correlation coefficient (*r*)	Rank	Stability value	Rank	Stability value
	4	ACTB	3.162	5	0.127	1	0.12	0.981	5	0.117	3	0.071
	1	B2M	1.316	1	0.081	3	0.20	0.999	1	0.017	1	0.035
	5	GAPDH	4.472	4	0.115	5	0.21	0.969	4	0.079	4	0.090
	6	HPRT1	6.000	6	0.152	6	0.28	0.997	6	0.143	5	0.111
	3	SDHA	2.711	3	0.096	2	0.15	0.999	3	0.06	2	0.059
	1	YWHAZ	2.000	2	0.093	4	0.20	0.987	2	0.036	1	0.035
L16	Rank	Genes	Geomean of ranking values	Rank	Average of SD	Rank	SD	Pearson correlation coefficient (*r*)	Rank	Stability value	Rank	Stability value
	3	ACTB	2.213	2	0.181	4	0.08	0.450	3	0.093	1	0.048
	6	B2M	6.000	6	0.360	6	0.31	0.946	6	0.373	6	0.224
	4	GAPDH	3.31	4	0.194	2	0.06	0.001	5	0.155	2	0.064
	5	HPRT1	3.976	5	0.215	5	0.14	0.911	2	0.090	4	0.146
	1	SDHA	1.861	1	0.179	3	0.07	0.032	4	0.123	1	0.048
	2	YWHAZ	1.861	3	0.193	1	0.03	0.001	1	0.090	3	0.100
L28	Rank	Genes	Geomean of	Rank	Average of SD	Rank	SD	Pearson correlation	Rank	Stability value	Rank	Stability value

			ranking values					coefficient (*r*)				
	4	ACTB	4.472	4	0.363	5	0.29	0.802	4	0.299	4	0.27
	2	B2M	1.861	2	0.241	3	0.15	0.913	2	0.051	1	0.051
	5	GAPDH	4.472	5	0.375	4	0.20	0.358	5	0.344	3	0.193
	3	HPRT1	2.280	3	0.280	1	0.10	0.799	3	0.142	2	0.137
	6	SDHA	6.000	6	0.409	6	0.43	0.988	6	0.362	5	0.317
	1	YWHAZ	1.189	1	0.232	2	0.13	0.963	1	0.026	1	0.051
L30	Rank	Genes	Geomean of ranking values	Rank	Average of SD	Rank	SD	Pearson correlation coefficient (*r*)	Rank	Stability value	Rank	Stability value
	4	ACTB	3.162	5	0.550	1	0.08	0.656	5	0.498	3	0.240
	2	B2M	2.060	3	0.384	2	0.26	0.896	3	0.221	1	0.073
	6	GAPDH	6.000	6	0.674	6	0.66	0.968	6	0.627	5	0.474
	1	HPRT1	1.565	1	0.348	3	0.29	0.962	2	0.073	1	0.073
	5	SDHA	4.472	4	0.539	5	0.66	0.987	4	0.420	4	0.374
	3	YWHAZ	2.213	2	0.349	6	0.31	0.999	1	0.069	2	0.139
L36	Rank	Genes	Geomean of ranking values	Rank	Average of SD	Rank	SD	Pearson correlation coefficient (*r*)	Rank	Stability value	Rank	Stability value
	5	ACTB	3.344	5	0.378	1	0.09	0.946	5	0.301	4	0.248
	1	B2M	1.732	1	0.268	3	0.31	0.999	1	0.071	2	0.118
	2	GAPDH	2.213	2	0.291	4	0.35	0.935	3	0.174	1	0.034
	4	HPRT1	2.828	4	0.327	2	0.19	0.898	2	0.160	3	0.214
	6	SDHA	6.000	6	0.586	6	0.58	0.849	6	0.560	5	0.361
	3	YWHAZ	2.783	3	0.314	5	0.36	0.911	4	0.225	1	0.034
L37	Rank	Genes	Geomean of ranking values	Rank	Average of SD	Rank	SD	Pearson correlation coefficient (*r*)	Rank	Stability value	Rank	Stability value
	5	ACTB	5.233	5	0.474	6	0.40	0.759	5	0.429	4	0.306
	3	B2M	2.449	3	0.344	1	0.19	0.981	3	0.109	3	0.255
	4	GAPDH	2.991	4	0.362	5	0.40	0.981	4	0.238	1	0.164
	2	HPRT1	2.213	2	0.336	2	0.36	0.924	2	0.096	2	0.222
	6	SDHA	5.422	6	0.600	4	0.40	0.408	6	0.578	5	0.404
	1	YWHAZ	1.316	1	0.308	3	0.39	0.998	1	0.082	1	0.164
L38	Rank	Genes	Geomean of ranking values	Rank	Average of SD	Rank	SD	Pearson correlation coefficient (*r*)	Rank	Stability value	Rank	Stability value
	1	ACTB	2.060	3	0.315	2	0.20	0.699	3	0.221	1	0.054
	3	B2M	2.378	1	0.294	4	0.24	0.926	2	0.173	3	0.266
	5	GAPDH	3.663	6	0.404	1	0.06	0.001	5	0.331	5	0.341
	2	HPRT1	2.340	2	0.296	5	0.28	0.984	1	0.164	2	0.248
	4	SDHA	2.632	4	0.355	3	0.20	0.551	4	0.266	1	0.054
	6	YWHAZ	5.477	5	0.401	6	0.33	0.691	6	0.357	4	0.310

L5

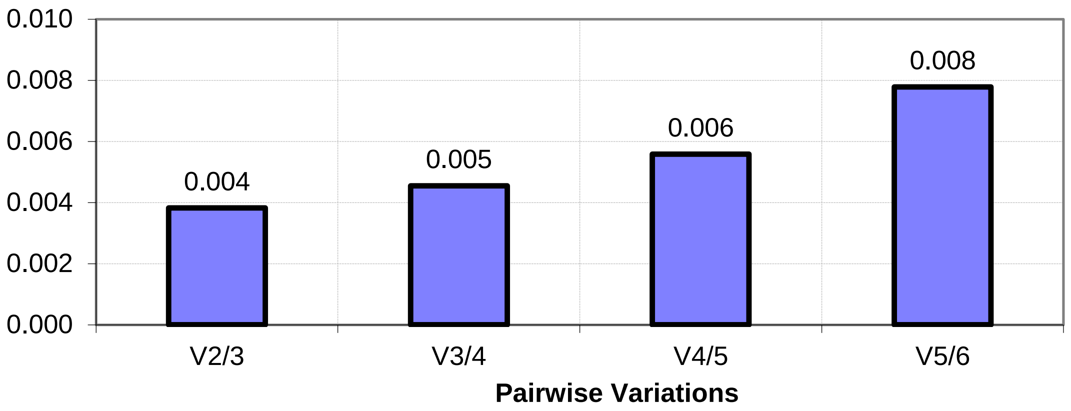
0.010
0.008
0.006
0.004
0.002
0.000
0.004
0.005
0.006
0.008
V2/3
V3/4
V4/5
V5/6
Pairwise Variations

L6

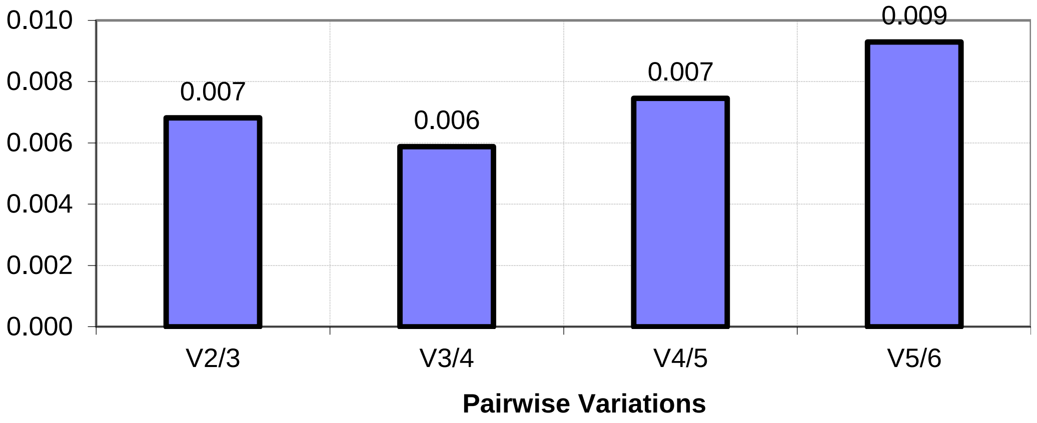
0.010
0.008
0.006
0.004
0.002
0.000
0.007
0.006
0.007
0.009
V2/3
V3/4
V4/5
V5/6
Pairwise Variations

L7

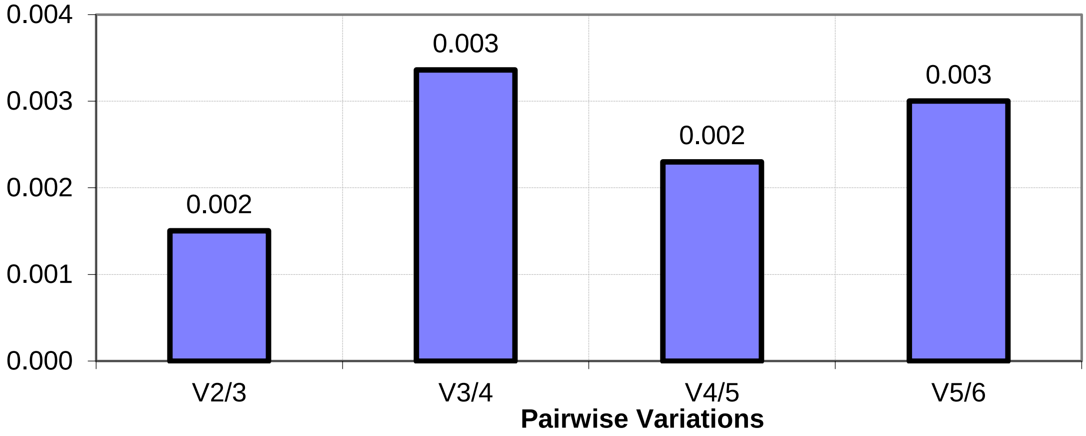
0.004
0.003
0.002
0.001
0.000
0.002
0.003
0.002
0.003
V2/3
V3/4
V4/5
V5/6
Pairwise Variations

L9

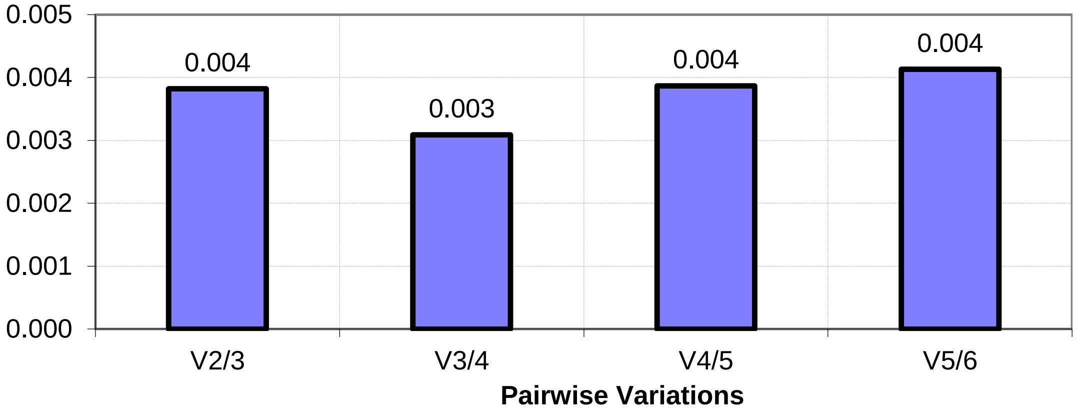
0.005
0.004
0.003
0.002
0.001
0.000
0.004
0.003
0.004
0.004
V2/3
V3/4
V4/5
V5/6
Pairwise Variations

L11

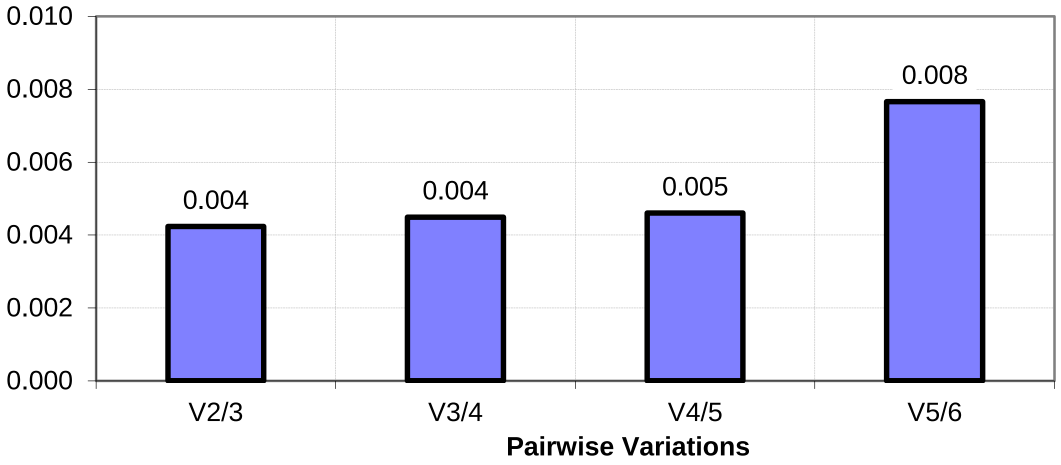

L14

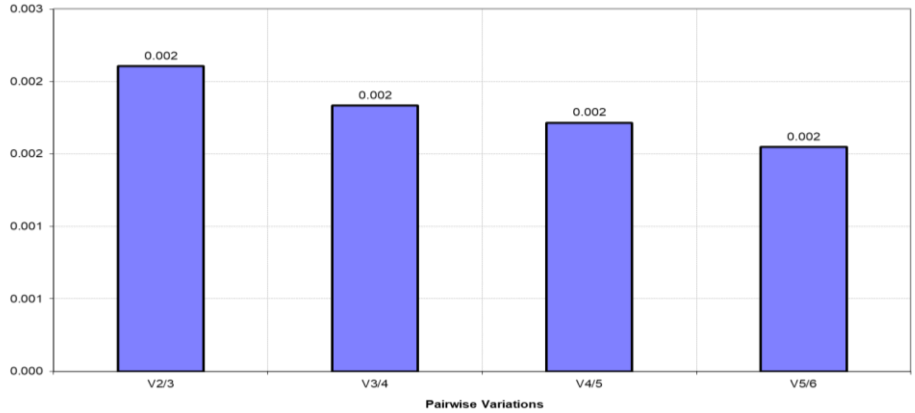

L16

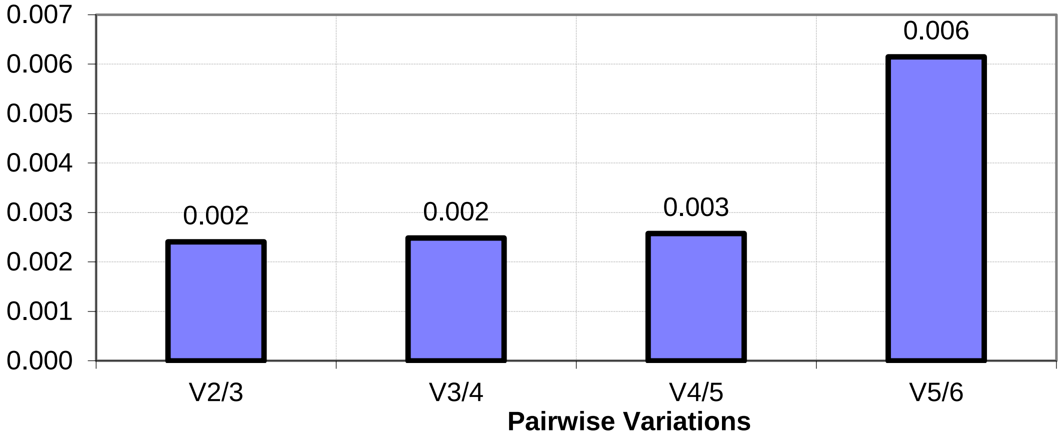

L28

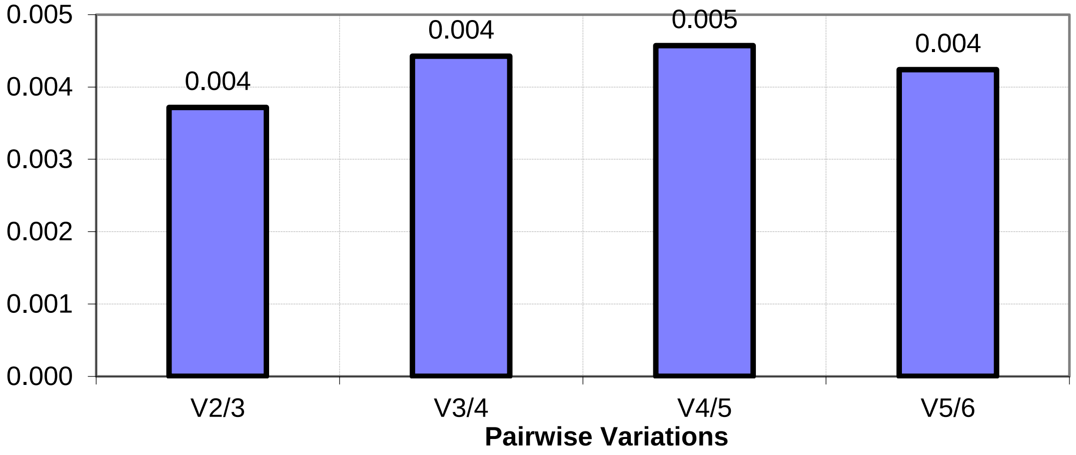

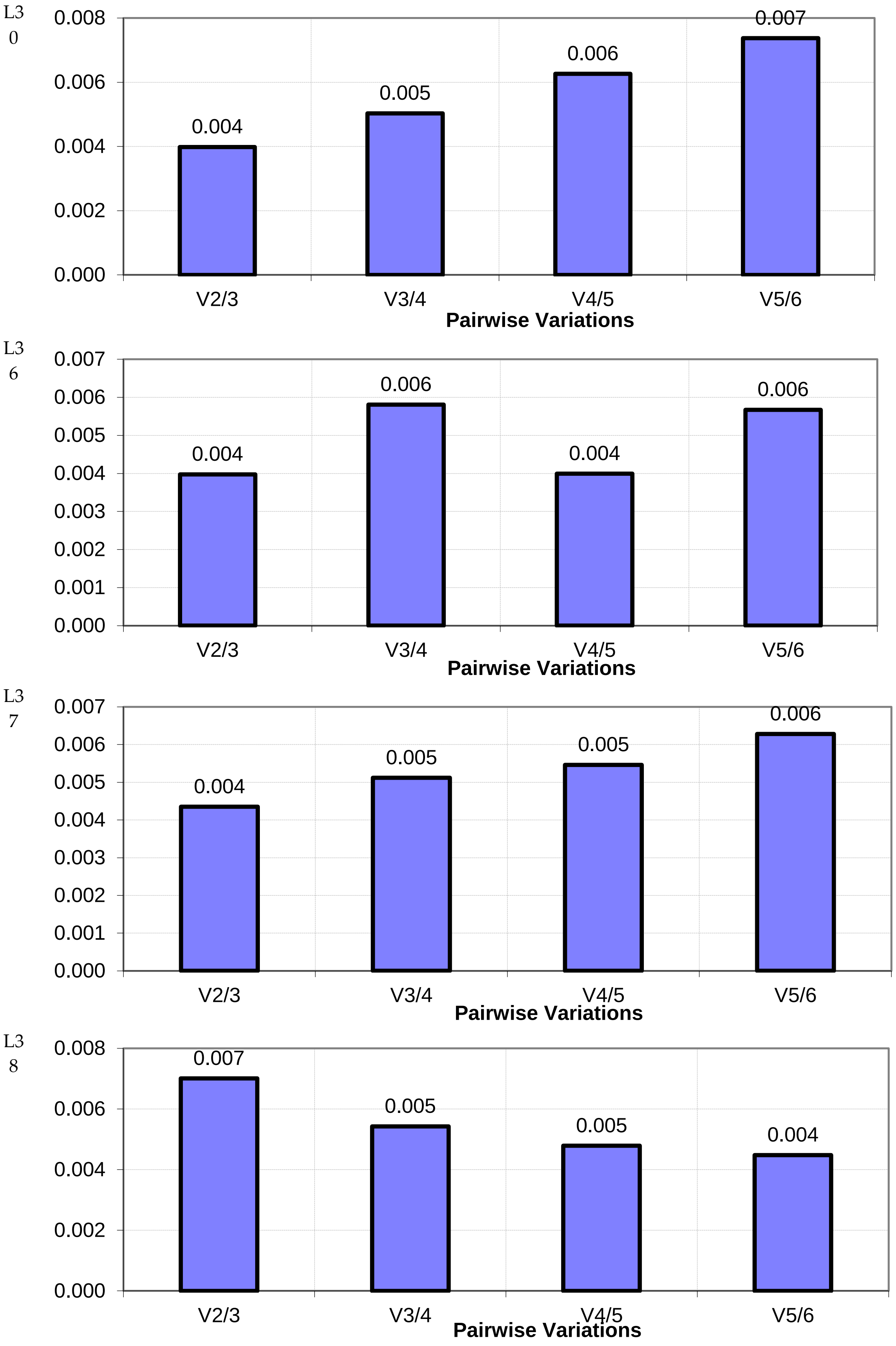

Figure S2. Determination of the optimal number of RGs in control PCLS by geNorm analysis

.

Table S7. RefFinder results for mRNA of treated PCLS incubated for 24 h with RIF/BNF/DMSO.

Sample No.	mRNA stability in human liver slices treated by DMSO+BNF+RIF											
	Comprehensive ranking			Delta CT		BestKeeper			NormFinder		geNorm	
L5	Rank	Genes	Geomean of ranking values	Rank	Average of SD	Rank	SD	Pearson correlation coefficient (*r*)	Rank	Stability value	Rank	Stability value
	1	ACTB	1.000	1	0.407	1	0.38	0.965	1	0.154	1	0.220
	3	B2M	3.310	4	0.534	2	0.43	0.834	5	0.427	2	0.321
	4	GAPDH	3.464	3	0.517	4	0.58	0.920	3	0.370	3	0.424
	5	HPRT1	4.949	5	0.554	6	0.71	0.944	4	0.427	4	0.476
	2	SDHA	1.861	2	0.456	3	0.44	0.919	2	0.279	1	0.220
	6	YWHAZ	5.733	6	0.563	5	0.64	0.922	6	0.453	5	0.505
L6	Rank	Genes	Geomean of ranking values	Rank	Average of SD	Rank	SD	Pearson correlation coefficient (*r*)	Rank	Stability value	Rank	Stability value
	1	ACTB	1.316	1	0.305	3	0.47	0.978	1	0.067	1	0.145
	5	B2M	4.729	5	0.457	4	0.47	0.819	5	0.386	4	0.349
	6	GAPDH	6.000	6	0.473	6	0.64	0.927	6	0.409	5	0.390
	4	HPRT1	4.229	4	0.398	5	0.62	0.949	4	0.285	3	0.305
	3	SDHA	2.280	3	0.363	1	0.39	0.938	3	0.218	2	0.233
	2	YWHAZ	1.682	2	0.346	2	0.44	0.934	2	0.211	1	0.145
L7	Rank	Genes	Geomean of ranking values	Rank	Average of SD	Rank	SD	Pearson correlation coefficient (*r*)	Rank	Stability value	Rank	Stability value
	1	ACTB	1.189	1	0.426	2	0.23	0.775	1	0.238	1	0.174
	6	B2M	6.000	6	0.673	6	0.64	0.834	6	0.628	5	0.505
	4	GAPDH	2.632	4	0.491	1	0.13	0.508	3	0.320	3	0.323
	5	HPRT1	5.000	5	0.511	5	0.50	0.899	5	0.351	4	0.422
	3	SDHA	2.632	2	0.461	4	0.30	0.683	2	0.299	2	0.253
	2	YWHAZ	2.449	3	0.470	3	0.29	0.662	4	0.348	1	0.174
L9	Rank	Genes	Geomean of ranking values	Rank	Average of SD	Rank	SD	Pearson correlation coefficient (*r*)	Rank	Stability value	Rank	Stability value
	5	ACTB	5.000	5	0.290	5	0.24	0.843	5	0.223	4	0.221
	3	B2M	2.376	4	0.256	2	0.14	0.549	4	0.197	1	0.103
	4	GAPDH	3.000	3	0.236	3	0.17	0.787	3	0.142	2	0.143
	1	HPRT1	1.189	1	0.221	1	0.13	0.756	2	0.113	1	0.103
	6	SDHA	6.000	6	0.351	6	0.27	0.535	6	0.317	5	0.265
	2	YWHAZ	2.378	2	0.233	4	0.17	0.841	1	0.103	3	0.186
L11	Rank	Genes	Geomean of ranking values	Rank	Average of SD	Rank	SD	Pearson correlation coefficient (*r*)	Rank	Stability value	Rank	Stability value
	1	ACTB	1.414	1	0.262	4	0.66	0.993	1	0.112	1	0.132
	3	B2M	2.913	3	0.300	2	0.54	0.987	3	0.164	3	0.226
	4	GAPDH	3.464	4	0.302	3	0.63	0.983	4	0.196	2	0.197
	2	HPRT1	2.115	2	0.278	5	0.67	0.995	2	0.143	1	0.132
	5	SDHA	3.834	6	0.476	1	0.52	0.898	6	0.444	5	0.326
	6	YWHAZ	5.233	5	0.338	6	0.74	0.975	5	0.259	4	0.251
L14	Rank	Genes	Geomean of ranking values	Rank	Average of SD	Rank	SD	Pearson correlation coefficient (*r*)	Rank	Stability value	Rank	Stability value
	2	ACTB	2.000	2	0.387	0.349	0.35	0.925	2	0.112	1	0.142
	6	B2M	6.000	6	0.692	0.693	0.69	0.910	6	0.657	6	0.474
	4	GAPDH	3.364	4	0.456	0.281	0.28	0.633	4	0.319	3	0.329

	5	HPRT1	5.000	5	0.516	0.375	0.38	0.466	5	0.439	4	0.365
	3	SDHA	2.280	3	0.426	0.104	0.10	0.778	3	0.245	2	0.257
	1	YWHAZ	1.316	1	0.364	0.318	0.32	0.955	1	0.071	1	0.142
L16	Rank	Genes	Geomean of ranking values	Rank	Average of SD	Rank	SD	Pearson correlation coefficient (*r*)	Rank	Stability value	Rank	Stability value
	2	ACTB	1.732	1	0.230	3	0.17	0.648	1	0.136	2	0.176
	6	B2M	6.000	6	0.449	6	0.25	0.419	6	0.410	5	0.321
	1	GAPDH	1.682	2	0.276	2	0.17	0.637	2	0.171	1	0.136
	5	HPRT1	4.000	4	0.313	4	0.18	0.385	4	0.209	3	0.217
	4	SDHA	3.344	5	0.330	1	0.16	0.489	5	0.231	4	0.257
	3	YWHAZ	2.590	3	0.284	6	0.21	0.844	3	0.179	1	0.136
L28	Rank	Genes	Geomean of ranking values	Rank	Average of SD	Rank	SD	Pearson correlation coefficient (*r*)	Rank	Stability value	Rank	Stability value
	6	ACTB	6.000	6	0.465	6	0.38	0.865	6	0.387	5	0.401
	1	B2M	1.000	1	0.309	1	0.23	0.931	1	0.060	1	0.120
	2	GAPDH	1.682	2	0.340	2	0.24	0.797	2	0.173	1	0.120
	4	HPRT1	3.722	4	0.439	3	0.25	0.458	4	0.355	3	0.300
	5	SDHA	4.729	5	0.445	4	0.28	0.689	5	0.359	4	0.368
	3	YWHAZ	3.409	3	0.406	5	0.30	0.667	3	0.311	2	0.251
L30	Rank	Genes	Geomean of ranking values	Rank	Average of SD	Rank	SD	Pearson correlation coefficient (*r*)	Rank	Stability value	Rank	Stability value
	3	ACTB	3.000	3	0.461	3	0.71	0.943	3	0.337	2	0.243
	5	B2M	4.000	4	0.472	4	0.73	0.935	4	0.365	3	0.263
	6	GAPDH	4.559	6	0.684	2	0.68	0.784	6	0.639	5	0.492
	2	HPRT1	1.861	2	0.400	6	0.77	0.985	1	0.154	1	0.206
	4	SDHA	3.344	5	0.544	1	0.61	0.902	5	0.411	4	0.397
	1	YWHAZ	1.778	1	0.394	5	0.74	0.976	2	0.165	1	0.206
L36	Rank	Genes	Geomean of ranking values	Rank	Average of SD	Rank	SD	Pearson correlation coefficient (*r*)	Rank	Stability value	Rank	Stability value
	4	ACTB	2.783	5	0.479	1	0.19	0.167	4	0.324	2	0.255
	3	B2M	2.340	3	0.443	2	0.20	0.766	1	0.108	4	0.292
	1	GAPDH	2.213	1	0.410	3	0.21	0.901	3	0.108	3	0.274
	2	HPRT1	2.213	2	0.437	4	0.21	0.553	4	0.208	1	0.229
	6	SDHA	6.000	6	1.078	6	0.76	0.406	6	1.063	5	0.554
	5	YWHAZ	3.162	4	0.475	5	0.34	0.639	5	0.336	1	0.229
L37	Rank	Genes	Geomean of ranking values	Rank	Average of SD	Rank	SD	Pearson correlation coefficient (*r*)	Rank	Stability value	Rank	Stability value
	5	ACTB	3.344	5	0.572	1	0.38	0.743	5	0.462	4	0.464
	2	B2M	2.632	2	0.456	4	0.47	0.933	2	0.222	2	0.351
	3	GAPDH	2.913	3	0.521	2	0.40	0.816	3	0.357	3	0.433
	4	HPRT1	3.313	4	0.531	6	0.52	0.931	4	0.401	1	0.300
	6	SDHA	5.045	6	0.648	3	0.44	0.546	6	0.562	5	0.526
	1	YWHAZ	1.495	1	0.425	5	0.49	0.959	1	0.130	1	0.300
L38	Rank	Genes	Geomean of ranking values	Rank	Average of SD	Rank	SD	Pearson correlation coefficient (*r*)	Rank	Stability value	Rank	Stability value
	3	ACTB	2.213	2	0.638	3	0.37	0.788	4	0.365	1	0.257
	2	B2M	1.861	3	0.644	1	0.34	0.722	1	0.257	3	0.405
	4	GAPDH	2.913	4	0.654	2	0.35	0.763	3	0.376	2	0.322
	1	HPRT1	1.682	1	0.619	4	0.43	0.882	2	0.348	4	0.257
	6	SDHA	5.733	6	1.037	5	0.50	0.001	6	0.918	6	0.762
	5	YWHAZ	5.223	5	0.976	6	0.60	0.593	5	0.837	5	0.624

L5

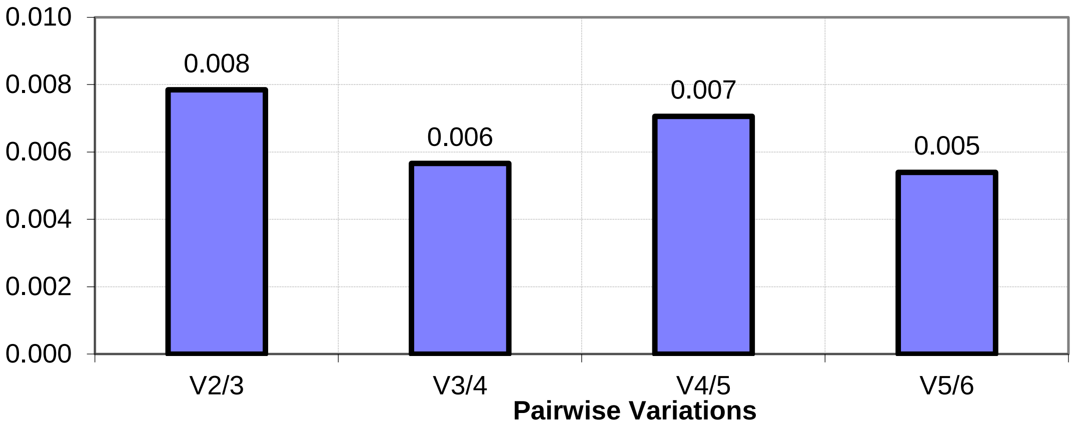
0.010
0.008
0.006
0.004
0.002
0.000
0.008
0.006
0.007
0.005
V2/3
V3/4
V4/5
V5/6
Pairwise Variations

L6

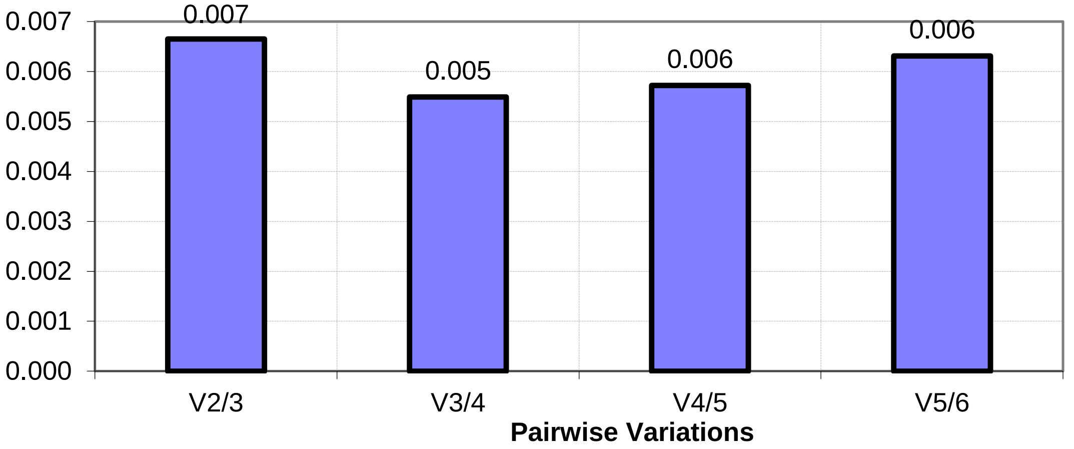
0.007
0.006
0.005
0.004
0.003
0.002
0.001
0.000
0.007
0.005
0.006
0.006
V2/3
V3/4
V4/5
V5/6
Pairwise Variations

L7

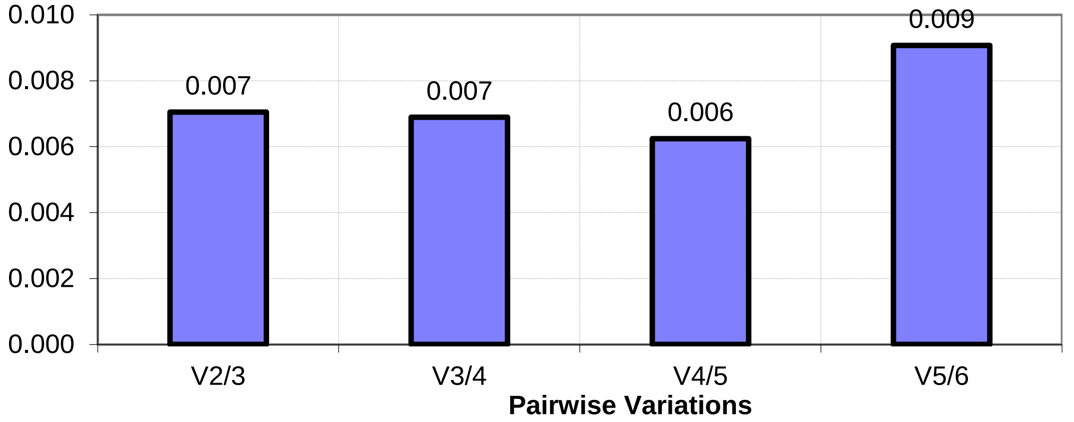
0.010
0.008
0.006
0.004
0.002
0.000
0.007
0.007
0.006
0.009
V2/3
V3/4
V4/5
V5/6
Pairwise Variations

L9

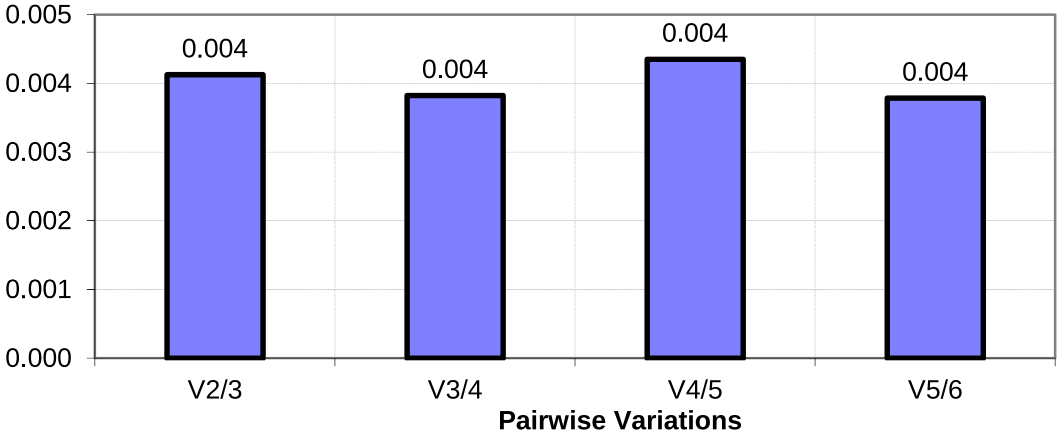
0.005
0.004
0.003
0.002
0.001
0.000
0.004
0.004
0.004
0.004
V2/3
V3/4
V4/5
V5/6
Pairwise Variations

L1
1

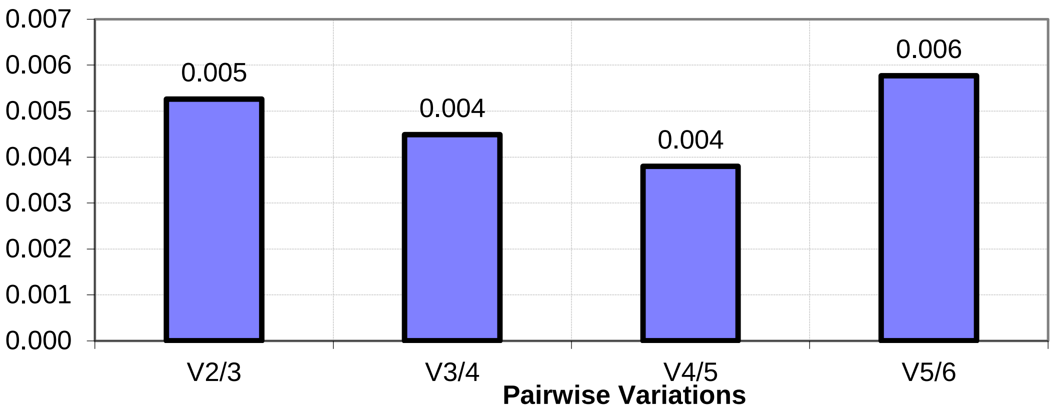

L1
4

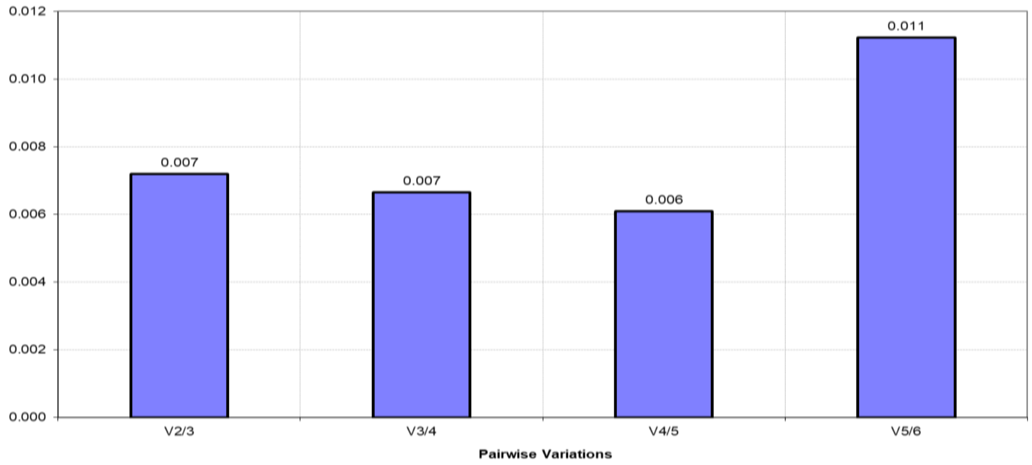

L1
6

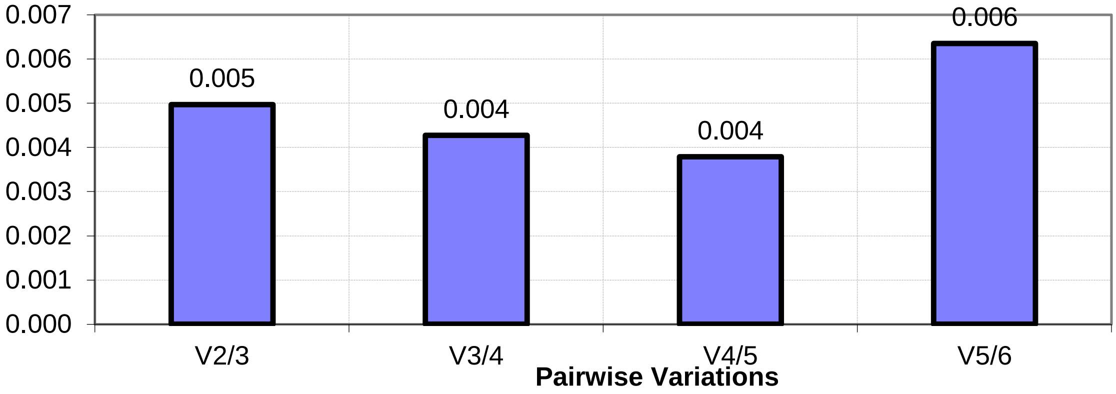

L2
8

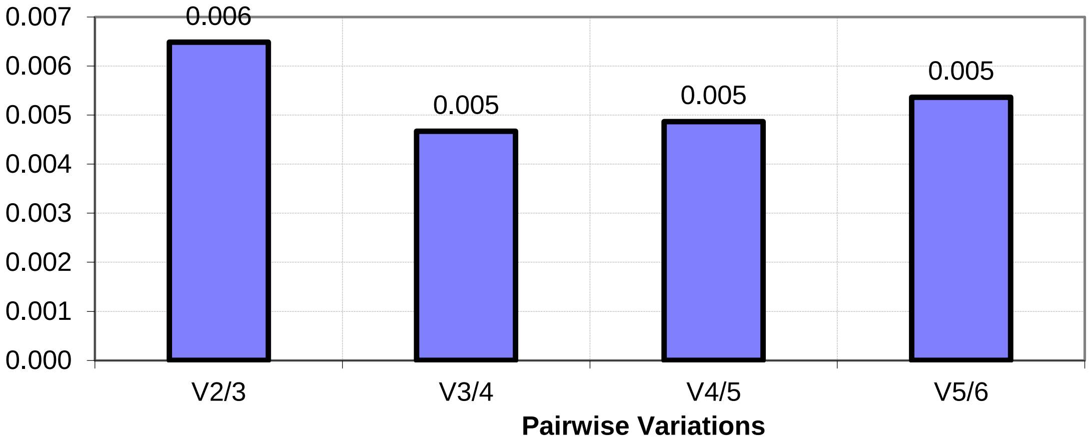

L30

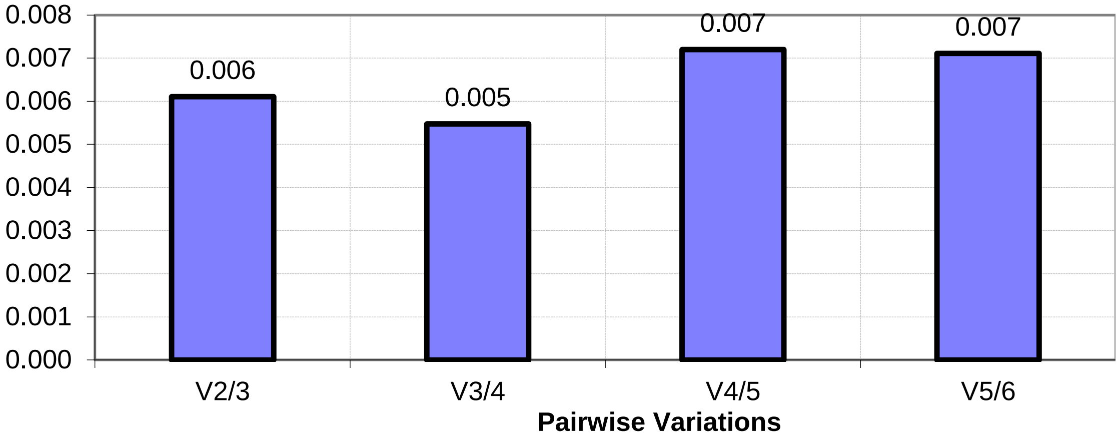

L36

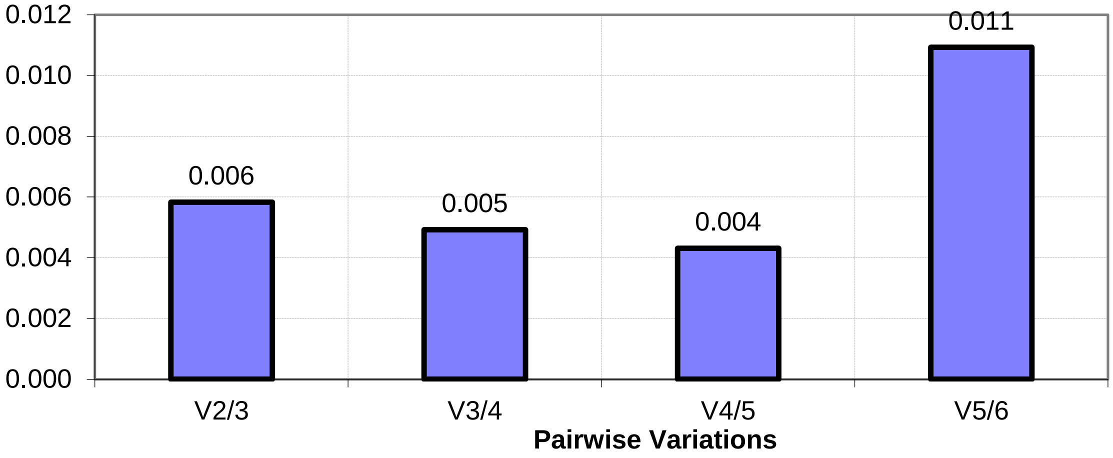

L37

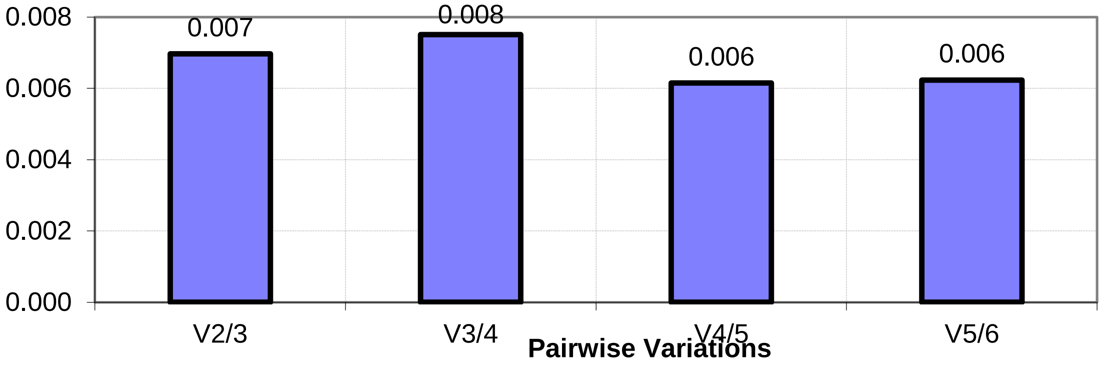

L38

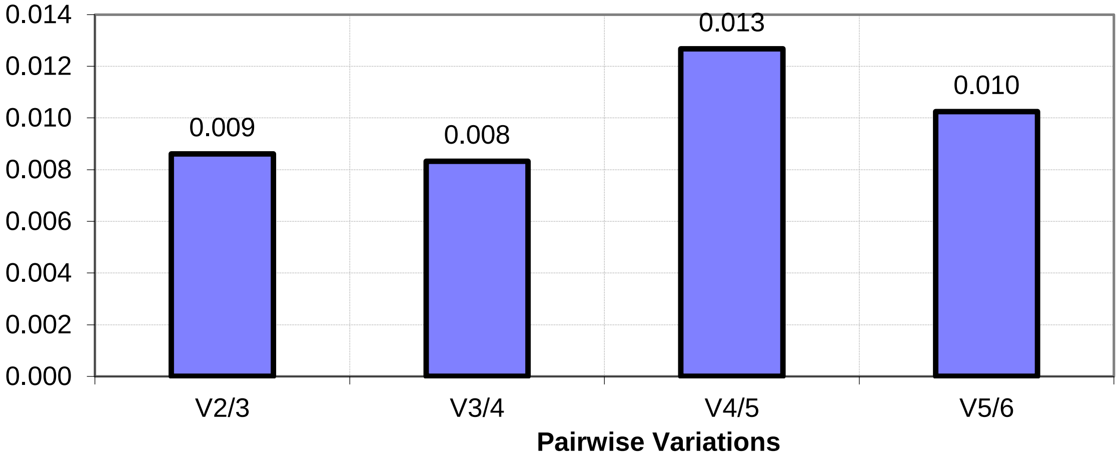

Figure S3. Determination of the optimal number of RGs in treated PCLS by geNorm analysis

Table S8. RefFinder results for mRNA of control PCLS incubated for 24 h with DMSO (time dependence).

	mRNA stability in human liver slices treated by DMSO												
	**Comprehensive ranking**			**Delta CT**		**BestKeeper**			**NormFinder**		**geNorm**	
	Rank	Genes	Geomean of ranking values	Rank	Average of SD	Rank	SD	Pearson correlation coefficient (*r*)	Rank	Stability value	Rank	Stability value
**L28**	2	**ACTB**	2.00	2	0.49	4	0.64	0.978	2	0.26	1	0.230
	1	**B2M**	1.19	1	0.45	2	0.59	0.960	3	0.27	2	0.293
	5	**GAPDH**	4.23	4	0.60	5	0.76	0.905	6	0.63	5	0.540
	6	**HPRT1**	4.40	5	0.63	3	0.60	0.920	4	0.40	3	0.371
	4	**SDHA**	3.83	6	0.64	1	0.46	0.896	5	0.47	4	0.453
	3	**YWHAZ**	3.57	3	0.50	6	0.81	0.982	1	0.17	1	0.233
	**Comprehensive ranking**			**Delta CT**		**BestKeeper**			**NormFinder**		**geNorm**	
	Rank	Genes	Geomean of ranking values	Rank	Average of SD	Rank	SD	Pearson correlation coefficient (*r*)	Rank	Stability value	Rank	Stability value
**L30**	**2**	**ACTB**	2.45	3	0.51	4	0.82	0.961	3	0.32	1	0.244
	**2**	**B2M**	2.45	2	0.48	3	0.70	0.967	2	0.20	3	0.283
	**6**	**GAPDH**	6.00	6	0.73	6	0.90	0.892	6	0.64	6	0.577
	**5**	**HPRT1**	3.36	4	0.56	2	0.65	0.918	4	0.36	4	0.376
	**4**	**SDHA**	3.34	5	0.72	1	0.48	0.792	5	0.62	5	0.499
	**1**	**YWHAZ**	1.50	1	0.46	5	0.83	0.989	1	0.18	1	0.244
	**Comprehensive ranking**			**Delta CT**		**BestKeeper**			**NormFinder**		**geNorm**	
	Rank	Genes	Geomean of ranking values	Rank	Average of SD	Rank	SD	Pearson correlation coefficient (*r*)	Rank	Stability value	Rank	Stability value
**L37**	**3**	**ACTB**	2.71	3	0.52	6	0.77	0.961	3	0.365	1	0.361
	**6**	**B2M**	4.40	5	0.61	3	0.51	0.810	5	0.477	5	0.508
	**5**	**GAPDH**	3.94	4	0.56	5	0.77	0.955	4	0.433	3	0.377
	**1**	**HPRT1**	1.68	1	0.48	2	0.50	0.931	1	0.228	4	0.421
	**4**	**SDHA**	3.83	6	0.63	1	0.46	0.748	6	0.530	6	0.549
	**2**	**YWHAZ**	2.00	2	0.49	4	0.75	0.969	2	0.291	1	0.361

L28

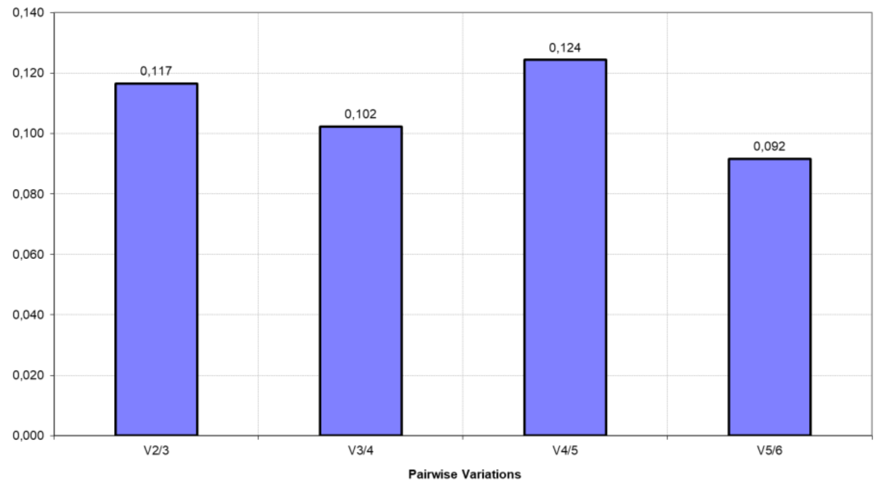

L30

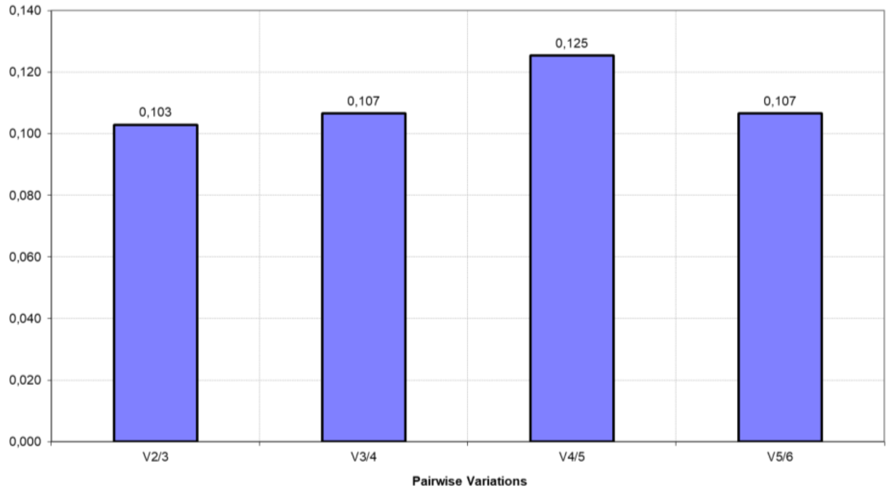

L37

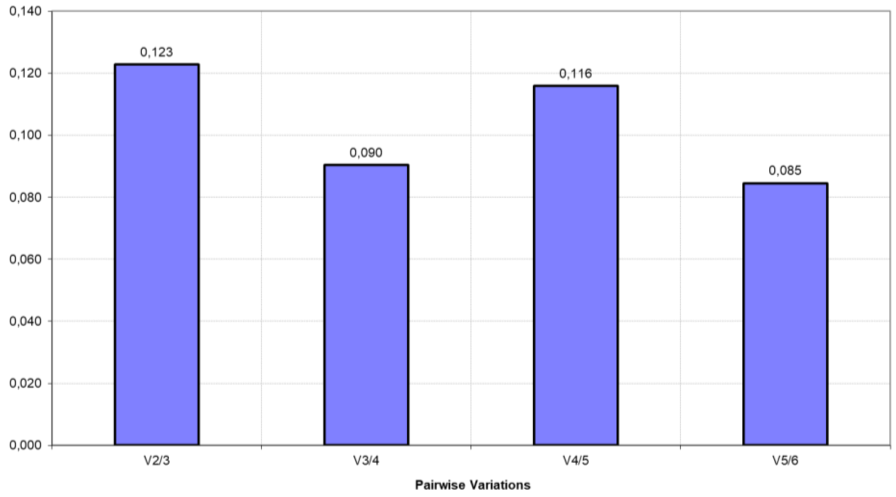

Figure S4. Determination of the optimal number of RGs in control PCLS by geNorm analysis.

Table S9. RefFinder results for mRNA of treated PCLS incubated for 24 h with RIF/BNF/DMSO (time dependence).

	mRNA stability in human liver slices treated by DMSO, BNF and RIF											
	**Comprehensive ranking**			**Delta CT**		**BestKeeper**			**NormFinder**		**geNorm**	
	Rank	Genes	Geomean of ranking values	Rank	Average of SD	Rank	SD	Pearson correlation coefficient (*r*)	Rank	Stability value	Rank	Stability value
**L28**	4	**ACTB**	3.83	3	0.57	6	0.71	0.92	3	0.431	4	0.432
	1	**B2M**	1.19	1	0.47	2	0.50	0.95	1	0.158	1	0.358
	5	**GAPDH**	3.94	4	0.58	4	0.68	0.92	5	0.448	3	0.406
	6	**HPRT1**	5.05	6	0.68	3	0.56	0.67	6	0.596	6	0.564
	3	**SDHA**	3.16	5	0.59	1	0.36	0.78	4	0.432	5	0.504
	2	**YWHAZ**	2.12	2	0.49	5	0.69	0.91	2	0.263	1	0.358
	**Comprehensive ranking**			**Delta CT**		**BesKkeeper**			**NormFinder**		**geNorm**	
	Rank	Genes	Geomean of ranking values	Rank	Average of SD	Rank	SD	Pearson correlation coefficient (*r*)	Rank	Stability value	Rank	Stability value
**L30**	2	**ACTB**	2.11	2	0.47	5	0.79	0.98	2	0.26	1	0.233
	3	**B2M**	2.28	3	0.47	1	0.60	0.96	3	0.27	3	0.293
	6	**GAPDH**	6.00	6	0.71	6	0.90	0.91	6	0.63	6	0.540
	4	**HPRT1**	3.72	4	0.55	3	0.70	0.92	4	0.40	4	0.371
	5	**SDHA**	3.98	5	0.60	2	0.61	0.90	5	0.47	5	0.453
	1	**YWHAZ**	1.41	1	0.43	4	0.75	0.98	1	0.17	1	0.23
	**Comprehensive ranking**			**Delta CT**		**BestKeeper**			**NormFinder**		**geNorm**	
	Rank	Genes	Geomean of ranking values	Rank	Average of SD	Rank	SD	Pearson correlation coefficient (*r*)	Rank	Stability value	Rank	Stability value
**L37**	6	**ACTB**	4.61	5	0.56	6	0.74	0.93	5	0.428	3	0.424
	4	**B2M**	3.56	4	0.56	2	0.52	0.86	4	0.399	5	0.489
	2	**GAPDH**	2.45	3	0.52	4	0.69	0.94	3	0.349	1	0.383
	3	**HPRT1**	2.63	2	0.49	3	0.56	0.92	2	0.280	4	0.446
	5	**SDHA**	3.83	6	0.64	1	0.47	0.73	6	0.547	6	0.540
	1	**YWHAZ**	1.50	1	0.47	5	0.72	0.97	1	0.261	2	0.383

L28

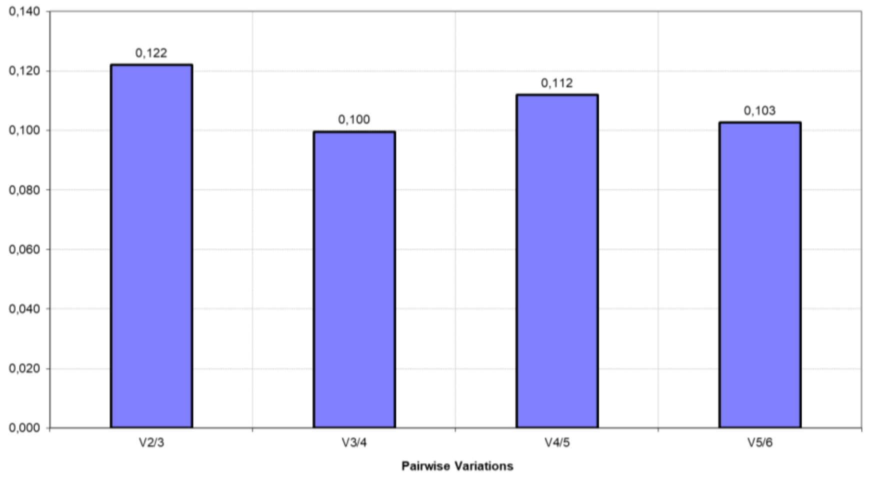

L30

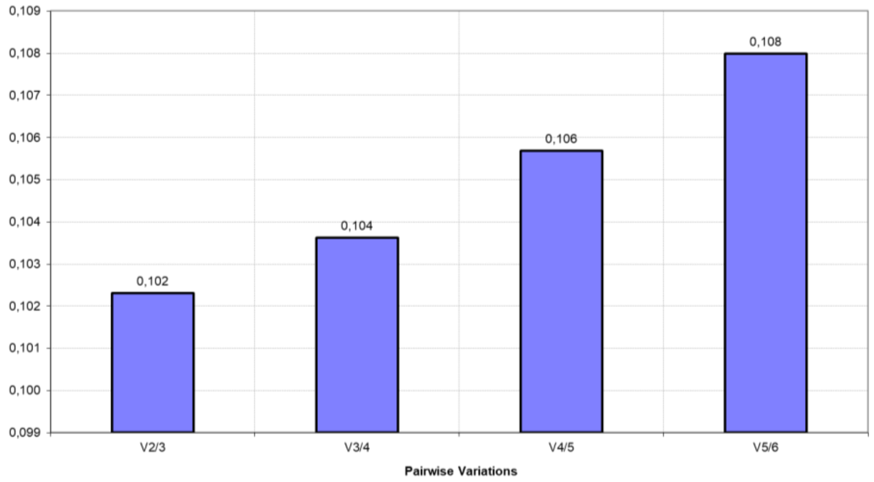

L37

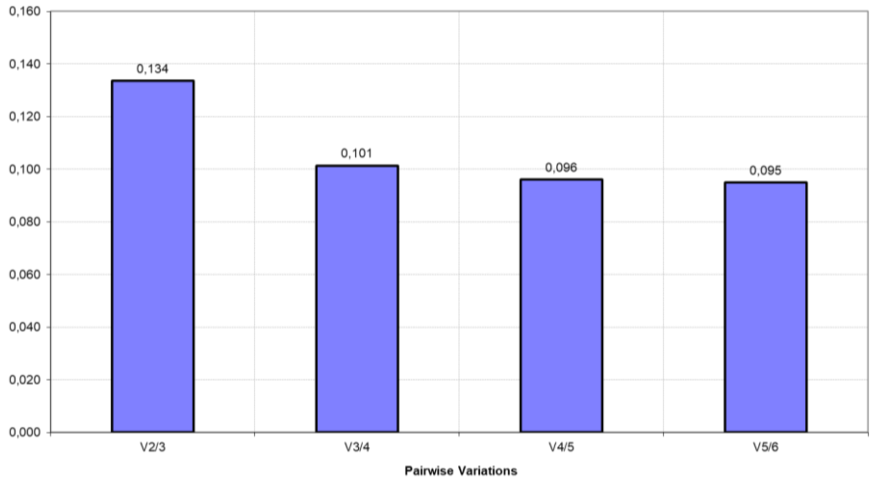

Figure S5. Determination of the optimal number of RGs in treated PCLS by geNorm analysis.

Table S10. RefFinder results for miRNA of control PCLS incubated for 24 h with DMSO (time dependence).

	miRNA stability in human liver slices treated by DMSO												
	**Comprehensive ranking**			**Delta CT**		**BestKeeper**			**NormFinder**		**geNorm**	
	Rank	Genes	Geomean of ranking values	Rank	Average of SD	Rank	SD	Pearson correlation coefficient (*r*)	Rank	Stability value	Rank	Stability value
**L28**	3	**miR-16-5p**	3.34	5	0.61	1	0.18	0.123	5	0.536	5	0.497
	5	**miR-23b-3p**	3.72	4	0.50	5	0.52	0.825	3	0.352	4	0.423
	4	**miR-93-5p**	3.66	3	0.50	4	0.55	0.963	4	0.370	3	0.384
	1	**miR-152-3p**	1.19	1	0.42	2	0.31	0.888	1	0.13	1	0.337
	2	**U6**	1.86	2	0.46	3	0.46	0.921	2	0.295	1	0.337
	**Comprehensive ranking**			**Delta CT**		**BestKeeper**			**NormFinder**		**geNorm**	
	Rank	Genes	Geomean of ranking values	Rank	Average of SD	Rank	SD	Pearson correlation coefficient (*r*)	Rank	Stability value	Rank	Stability value
**L30**	2	**miR-16-5p**	1.68	2	0.28	2	0.18	0.688	2	0.181	1	0.222
	3	**miR-23b-3p**	3.46	4	0.32	3	0.18	0.426	3	0.246	4	0.286
	1	**miR-93-5p**	1.00	1	0.26	1	0.13	0.751	1	0.114	1	0.222
	5	**miR-152-3p**	5.00	5	0.32	5	0.27	0.751	5	0.251	5	0.300
	4	**U6**	3.46	3	0.32	4	0.23	0.732	4	0.246	3	0.253
	**Comprehensive ranking**			**Delta CT**		**BestKeeper**			**NormFinder**		**geNorm**	
	Rank	Genes	Geomean of ranking values	Rank	Average of SD	Rank	SD	Pearson correlation coefficient (*r*)	Rank	Stability value	Rank	Stability value
**L37**	2	**miR-16-5p**	1.57	2	0.30	1	0.20	0.748	3	0.210	1	0.109
	3	**miR-23b-3p**	2.45	3	0.30	3	0.23	0.824	1	0.163	4	0.261
	5	**miR-93-5p**	5.00	5	0.42	5	0.41	0.780	5	0.377	5	0.325
	1	**miR-152-3p**	1.41	1	0.29	2	0.21	0.780	2	0.168	1	0.109
	4	**U6**	3.72	4	0.32	4	0.25	**0.834**	4	0.221	3	0.235

L28

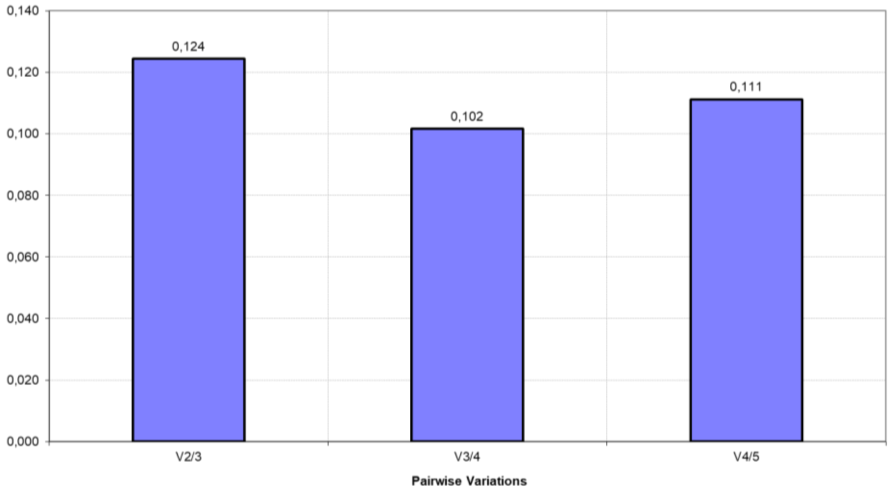

L30

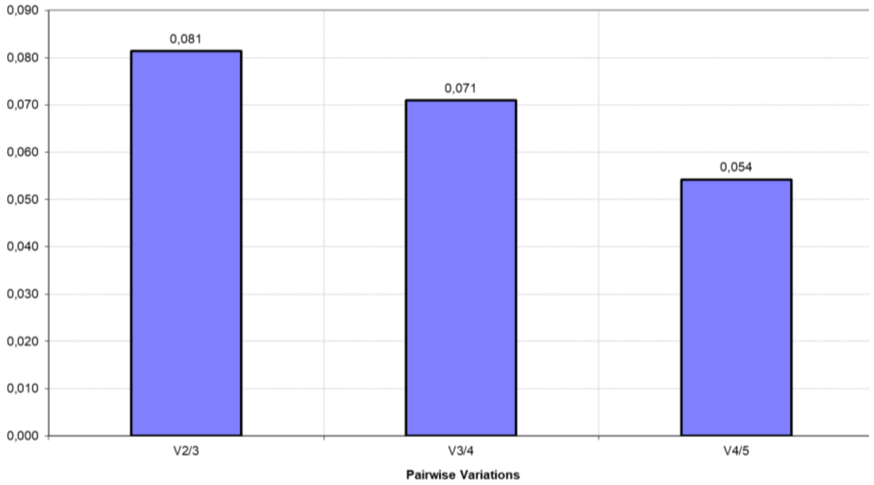

L37

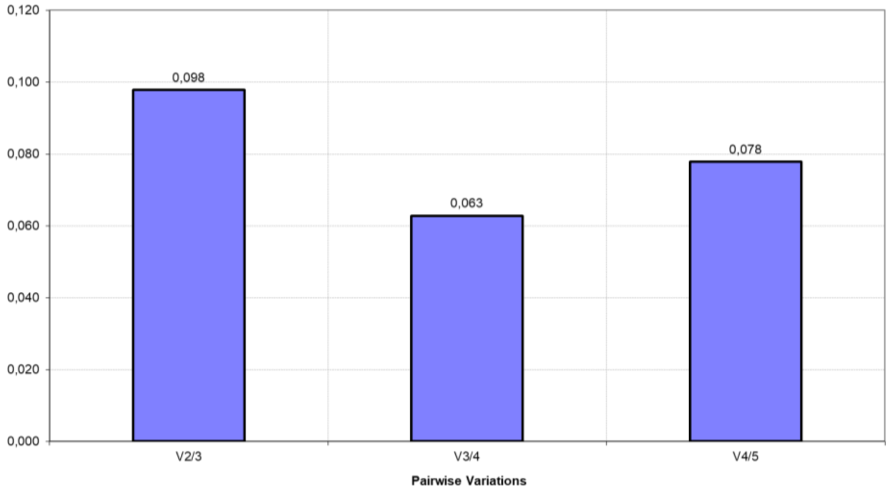

Figure S6. Determination of the optimal number of RGs in control PCLS by geNorm analysis.

Table S11. RefFinder results for miRNA of treated PCLS incubated for 24 h with RIF/BNF/DMSO (time dependence).

miRNA stability in human liver slices treated by DMSO, BNF and RIF												
	**Comprehensive ranking**			**Delta CT**		**BestKeeper**			**NormFinder**		**geNorm**	
	Rank	Genes	Geomean of ranking values	Rank	Average of SD	Rank	SD	Pearson correlation coefficient (*r*)	Rank	Stability value	Rank	Stability value
**L28**	3	**miR-16-5p**	2.83	4	0.50	1	0.19	0.195	4	0.408	4	0.431
	1	**miR-23b-3p**	1.41	1	0.43	4	0.35	0.804	1	0.248	1	0.385
	5	**miR-93-5p**	5.00	5	0.51	5	0.43	0.802	5	0.419	5	0.463
	2	**miR-152-3p**	1.68	2	0.43	2	0.26	0.648	2	0.255	1	0.385
	4	**U6**	3.00	3	0.44	3	0.27	0.831	3	0.285	3	0.404
	**Comprehensive ranking**			**Delta CT**		**BestKeeper**			**NormFinder**		**geNorm**	
	Rank	Genes	Geomean of ranking values	Rank	Average of SD	Rank	SD	Pearson correlation coefficient (*r*)	Rank	Stability value	Rank	Stability value
**L30**	1	**miR-16-5p**	1.73	3	0.36	1	0.2	0.535	3	0.236	1	0.303
	1	**miR-23b-3p**	1.73	1	0.34	3	0.21	0.733	1	0.169	3	0.32
	3	**miR-93-5p**	2.38	2	0.36	2	0.2	0.634	2	0.23	4	0.323
	5	**miR-152-3p**	5.00	5	0.45	5	0.34	0.645	5	0.395	5	0.375
	4	**U6**	2.83	4	0.37	4	0.21	0.673	4	0.268	1	0.303
	**Comprehensive ranking**			**Delta CT**		**BestKeeper**			**NormFinder**		**geNorm**	
	Rank	Genes	Geomean of ranking values	Rank	Average of SD	Rank	SD	Pearson correlation coefficient (*r*)	Rank	Stability value	Rank	Stability value
**L37**	2	**miR-16-5p**	1.41	2	0.41	1	0.24	0.721	2	0.217	1	0.169
	3	**miR-23b-3p**	3.46	4	0.5	3	0.31	0.503	3	0.378	4	0.406
	4	**miR-93-5p**	3.46	3	0.5	4	0.36	0.535	4	0.383	3	0.351
	1	**miR-152-3p**	1.19	1	0.38	2	0.25	0.780	1	0.134	1	0.169
	5	**U6**	5.00	5	0.57	5	0.36	0.857	5	0.493	5	0.472

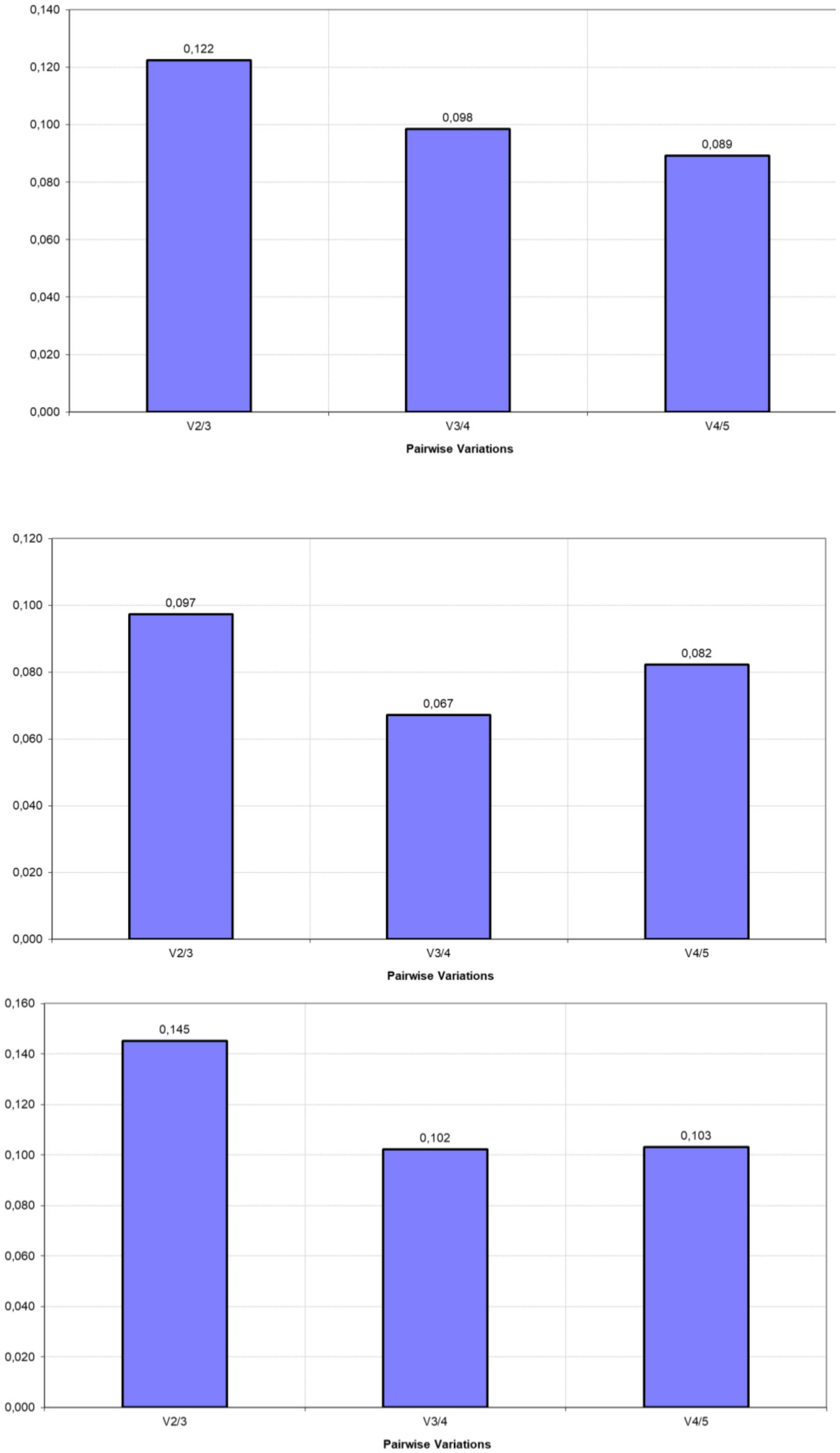

Figure S7. Determination of the optimal number of RGs in treated PCLS by geNorm analysis.
